# New Acceptor–Donor–Acceptor Systems Based on Bis-(Imino-1,8-Naphthalimide)

**DOI:** 10.3390/ma14112714

**Published:** 2021-05-21

**Authors:** Sonia Kotowicz, Mateusz Korzec, Agnieszka Katarzyna Pająk, Sylwia Golba, Jan Grzegorz Małecki, Mariola Siwy, Justyna Grzelak, Sebastian Maćkowski, Ewa Schab-Balcerzak

**Affiliations:** 1 Institute of Chemistry, University of Silesia, 9 Szkolna Str., 40-006 Katowice, Poland; agpajak@us.edu.pl (A.K.P.); jan.malecki@us.edu.pl (J.G.M.); ewa.schab-balcerzak@us.edu.pl (E.S.-B.); 2 Institute of Materials Engineering, University of Silesia, 75 Pulku Piechoty Str., 41-500 Chorzow, Poland; sylwia.golba@us.edu.pl; 3 Centre of Polymer and Carbon Materials, Polish Academy of Sciences, 34 M. Curie-Sklodowska Str., 41-819 Zabrze, Poland; msiwy@cmpw-pan.edu.pl; 4 Faculty of Physics, Institute of Physics, Astronomy and Informatics, Nicolaus Copernicus University, 5 Grudziadzka Str., 87-100 Torun, Poland; justynag@fizyka.umk.pl (J.G.); mackowski@fizyka.umk.pl (S.M.)

**Keywords:** 1,8-naphthalimides, C-3 position, azomethinediimides, electrochemistry, luminescence

## Abstract

In this paper, six novel symmetrical bis-(imino-1,8-naphthalimides) differing in core and N-substituent structure were synthesized, and their thermal (TGA, DSC), optical (UV-Vis, PL), electrochemical (DPV, CV) properties were evaluated. The compounds were stable to 280 °C and could be transferred into amorphous materials. Electrochemical investigations showed their ability to occur reductions and oxidations processes. They exhibited deep LUMO levels of about −3.22 eV and HOMO levels above −5.80 eV. The optical investigations were carried out in the solutions (polar and non-polar) and in films and blends with PVK:PBD. Bis-(imino-1,8-naphthalimides) absorbed electromagnetic radiation in the range of 243–415 nm and emitted light from blue to yellow. Their capacity for light emission under voltage was preliminarily tested in devices with an active layer consisting of a neat compound and a blend with PVK:PBD. The diodes emitted green or red light.

## 1. Introduction

The discovery of organic compounds with semiconductor properties has opened up new possibilities for the development of electronics. A significant advantage of organic semiconductors is the practically unlimited possibility of modifying their physicochemical properties by appropriately designing their chemical structure. The imide rings as *n*-type semiconductors are very popular as electron acceptors with the possibility of functionalizing [1,2,3,4]. Due to their structure, imide rings can be divided into five- and six-membered rings obtained from phthalic or naphthalene/perylene anhydrides. Many publications associated with imide rings in organic electronics include symmetrical structures (phthalic diimides, naphthalene, perylene) [5,6,7,8,9,10]. The imide molecules’ properties can be changed by modifying the substituent type attached to the nitrogen atom or, more broadly, by modifying the perylene or naphthalene ring (4-C or 3-C) [9]. The introduction of structures with electron-donating properties to the naphthalene ring allows the obtainment of materials with a low band gap, which is beneficial in optoelectronic applications [11]. Compounds containing imide rings are characterized by many valuable properties, i.e., high thermal, chemical/oxidation resistance and the ability to self-organize [9]. Imides obtained from naphthalene anhydride as derivatives of 1,8-naphthalimide are widely used in medicine and biology, as compounds with high antitumor activity, cell dyes for bioimaging [12,13,14] and as ion detectors [15]. Most compounds containing 1,8-naphthalimide show absorption and emission in the visible spectrum region and good photo- and thermal-stability [16,17,18,19,20,21,22]. 1,8-Naphthalimide derivatives in OLEDs and OPVs have been studied as electron-transporting materials [23,24], emitters [25,26,27,28,29,30,31], emission polymers additionally transporting electrons [32], as a host in the guest-host structure (acting as a matrix for the blue emitter) [9], and as green dopant [33]. 1,8-Naphthalimides showed high electron mobility [34], and further the presence of a donor substituent allows to obtain compounds with dominant transport of holes and higher emission intensity [35,36,37,38] and high electron affinity [39]. Asymmetrical 1,8-naphthalimides structures, constructed from a single imide ring, are often tested as compounds substituted in the 4-C position for organic electronics [1,39,40]. In the papers [8,9,41,42,43,44] the thermal, optical, and electrochemical properties of azomethinediimides, as potential candidates for optoelectronic were presented. To the best of our knowledge, only few papers present naphthalimide derivatives with imine bond substituted in the 4-C or 3-C position investigated towards applications in organic electronics [1,45,46]. 

In the Supplementary Information in Figure S1 (see ESI) the structures of bisnaphthalimide derivatives are presented, dividing them according to the type of linker between 1,8-naphthalimide unit and the core of the compound. The described bisnaphthalimide derivatives include symmetric donor-acceptor structures with linkers, such as, vinyl [47], ethynyl [36], azo- [39,48], imino- [45], C-S [49] or C-O [50] bonds as well as without linkers by directly C–C binding with aryl [38,51]. Moreover, the bisnaphthalimides described so far in their core contained the following derivatives: phenyl, carbazole, triphenylamine, thiophene or bitiophene (Figure S1) [51]. To bisnaftaloimisdes are also include structures through the imide part [52,53,54] as well as through a bridge, e.g., amide [55,56]. Should be noted that the current research focuses on the synthesis and study of derivatives substituted in the 4-C position of the naphthalimide ring [51] while there are few reports on derivatives substituted in the 3-C position of the naphthalimide [45], as was mentioned above. In Table S1 (in ESI) collected data from optical and electrochemical measurements for the selected compounds shown in Figure S1 based on the available literature. 

Herein, the synthesis of new six symmetrical bis-(imino-1,8-naphthalimides) formed the A–π–D–π–A system and its characterization are reported. The study is focused on impact of donor and N-substituent structure on their thermal, photophysical, and electrochemical properties. The research was supplemented with the Density Functional Theory (DFT) calculations. Additionally, the preliminary attempts for applications of obtained bis-(imino-1,8-naphthalimides) in OLEDs are presented.

## 2. Materials, Methods and Synthesis 

Related information to measurements, films and device preparations as well as DFT calculations are given in the ESI. Describe of the synthesis is also given in the ESI. 

### 2.1. Materials

Dialdehydes, TFA, Pd/C, 3-nitro-1,8-naphthalic anhydride, hydrazine, hexylamine, benzylamine, 4-methylbenzylamine, 2-phenethylamine, triethylamine (99%), PVK, PBD, Bu_4_NPF_6_, NaOH, PPh_3_, Pd(PPh_3_)_2_Cl_2_ were purchased from Sigma Aldrich (Merck). Trimethylsilylacetyle (98%) and 4-bromobenzaldehyde (99%) were purchased from Acros Organic. Solvents were purchased from Sigma Aldrich (Merck) and Avantor S.A. PEDOT:PSS and glass with ITO surface were purchased from OSSILA.

### 2.2. Structural Characterization

5,5′-(triphenylamine-4,4′-diimine)-bis(2-(2-hylhexyl)-1H-benzo[de]isoquinoline-1,3(2H)-dione) (1a)

Yellow solid; Yield= 75%, ^1^H NMR (δ, 400 MHz, CDCl_3_, ppm): 8.63 (s, 2H), 8.55–8.52 (m, 4H), 8.18 (d, J = 8.1 Hz, 2H), 7.95 (d, J = 1.8 Hz, 2H), 7.90 (d, J = 8.6 Hz, 4H), 7.74 (t, J = 7.8 Hz, 2H), 7.42 (t, J = 7.6 Hz, 2H), 7.29–7.22 (m, 7H), 4.25–4.16 (m, 4H), 1.83–1.70 (m, 4H), 1.51–1.29 (m, 12H), 0.91 (t, J = 7.0 Hz, 6H). ^13^C NMR (δ, 101 MHz, CDCl_3_, ppm): 164.13, 164.03, 161.15, 150.66, 150.39, 146.15, 133.54, 132.68, 130.49, 130.11, 129.92, 127.38, 127.20, 126.5, 126.39, 125.43, 125.17, 124.62, 123.77, 123.26, 122.76, 40.58, 31.57, 28.09, 26.81, 22.58, 14.07. Anal. Calcd for C_56_H_51_N_5_O_4_ (858.04 g/mol): C (78.39%), H (5.99%), N (8.16%), Found: C (78.08%), H (5.91%), N (7.96%).

5,5′-(triphenylamine-4,4′-diimine)-bis(2-(2-benzyl)-1H-benzo[de]isoquinoline-1,3(2H)-dione (2a)

Light brown solid; Yield= 45%, ^1^H NMR (δ, 400 MHz, CDCl_3_, ppm): 8.62 (s, 2H), 8.58–8.53 (m, 4H), 8.20 (d, J = 8.1 Hz, 2H), 7.98 (d, J = 1.8 Hz, 2H), 7.90 (d, J = 8.6 Hz, 4H), 7.75 (t, J = 7.8 Hz, 2H), 7.59 (d, J = 7.2 Hz, 4H), 7.42 (t, J = 7.8 Hz, 2H), 7.34 (t, J = 7.4 Hz, 4H), 7.29–7.25 (m, 9H), 5.41 (s, 4H). ^13^C NMR (δ, 101 MHz, CDCl_3_, ppm): insufficient concentration. Anal. Calcd for C_58_H_39_N_5_O_4_ (869.07 g/mol): C (80.07%), H (4.52%), N (8.05%), Found: C (79.78%), H (4.47%), N (8.07%).

5,5′-(triphenylamine-4,4′-diimine)-bis(2-(2-metylobenzyl)-1H-benzo[de]isoquinoline-1,3(2H)-dione) (3a)

Light brown solid; Yield= 69%,^1^H NMR (δ, 400 MHz, CDCl_3_ppm): 8.62 (s, 2H), 8.58–8.53 (m, 4H), 8.20 (d, J = 8.3 Hz, 2H), 7.99 (d, J = 1.7 Hz, 2H), 7.90 (d, J = 8.6 Hz, 4H), 7.76 (m, 2H), 7.49 (d, J = 7.9 Hz, 4H), 7.41 (t, J = 7.8 Hz, 2H), 7.31–7.23 (m, 7H), 7.14 (d, J = 7.8 Hz, 4H), 5.39 (s, 4H), 2.33 (s, 6H). ^13^C NMR (δ, 101 MHz, CDCl_3_, ppm): 164.28, 164.13, 161.21, 150.69, 150.42, 146.15, 137.16, 134.34, 133.75, 132.72, 130.46, 130.38, 129.92, 129.11, 129.00, 127.41, 126.83, 126.51, 126.47, 125.42, 125.33, 124.98, 123.76, 123.27, 122.74, 43.39, 21.12. Anal. CalcdforC_60_H_43_N_5_O_4_ (898.01 g/mol): C (80.25%), H (4.83%), N (7.80%), Found: C (79.93%), H (4.67%), N (7.94%).

5,5′-(thiophene-2,5-diimine)-bis(2-(2-phenethyl)-1H-benzo[de]isoquinoline-1,3(2H)-dione) (4b)

Light brown solid; Yield= 69%,*^1^H NMR* (δ, 400 MHz, CDCl_3_ppm): 8.87 (s, 2H), 8.65–8.55 (m, 4H), 8.25 (d, *J* = 8.2 Hz, 2H), 8.09 (m, 2H), 7.80 (t, *J* = 7.7 Hz, 2H), 7.67 (s, 2H), 7.41 (m, 4H), 7.35 (t, *J* = 7.4 Hz, 4H), 7.30–7.23 (m, 2H), 4.43 (m, 4H), 3.13–2.97 (m, 4H). *^13^C NMR* (δ, 101 MHz, CDCl_3_, ppm): 163.92, 163.81, 154.14, 149.13, 146.62, 138.77, 133.91, 133.14, 132.65, 130.64, 129.03, 128.52, 127.65, 126.83, 126.49, 126.06, 124.42, 123.86, 122.74, 41.86, 34.31.Anal. Calcd for C_46_H_32_N_4_O_4_S (736.84 g/mol): C (74.98%), H (4.38%), N (7.60%), Found: C (79.72%), H (4.43%), N (7.56%).

5,5′-(biphenyl-4,4′-diimine)-bis(2-(2-hylhexyl)-1H-benzo[de]isoquinoline-1,3(2H)-dione) (1c)

Yellow solid; Yield= 65%, ^1^H NMR (δ, 400 MHz, CDCl_3_, ppm): 8.74 (s, 2H), 8.60–8.52 (m, 4H), 8.22 (d, J = 7.9 Hz, 2H), 8.10 (d, J = 8.2 Hz, 4H), 8.02 (d, J = 1.6 Hz, 2H), 7.84 (d, J = 8.2 Hz, 4H), 7.77 (t, J = 7.7 Hz, 2H), 4.28–4.14 (m, 4H), 1.85–1.70 (m, 4H), 1.54–1.29 (m, 12H), 0.92 (t, J = 6.8 Hz, 6H). ^13^C NMR (δ, 101 MHz, CDCl_3_, ppm): 164.02, 161.53, 150.32, 143.46, 135.39, 133.62, 132.61, 130.35, 129.76, 127.65, 127.46, 126.54, 125.01, 124.78, 123.83, 122.77, 40.60, 31.56, 28.09, 26.80, 22.57, 14.06. Anal. Calcd for C_50_H_46_N_4_O_4_ (766.92 g/mol): C (78.30%), H (6.05%), N (7.31%), Found: C (77.90%), H (5.99%), N (7.34%).

5,5′-(ethyne-1,2-diyl-4,4′-diimine)-bis(2-(2-hylhexyl)-1H-benzo[de]isoquinoline-1,3(2H)-dione) (1d)

Yellow solid; Yield= 55%. ^1^H NMR (δ, 400 MHz, CDCl_3_, ppm): 8.70 (s, 2H), 8.57 (d, J = 8.3 Hz, 4H), 8.23 (d, J = 8.0 Hz, 2H), 8.03–7.96 (m, 6H), 7.79 (t, J = 7.7 Hz, 2H), 7.71 (d, J = 8.1 Hz, 4H), 4.28–4.10 (m, 4H), 1.82–1.67 (m, 4H), 1.50–1.25 (m, 12H), 0.91 (m, 6H). ^13^C NMR (δ, 101 MHz, CDCl_3_, ppm): 164.43, 160.89, 149.94, 145.09, 136.27, 133.70, 133.09, 131.61, 129.57, 129.10, 127.56, 127.23, 125.01, 124.78, 122.84, 121.99, 114.04, 40.54, 31.57, 28.08, 26.72, 22.57, 14.05. Anal. Calcd for C_52_H_46_N_4_O_4_ (790.95 g/mol)C (78.96%), H (5.86%), N (7.08%), Found: C (78.86%) H (5.76%), N (6.93%).

## 3. Result and Discussion

### 3.1. Synthesis and Structural Characterization

**Figure 1:**
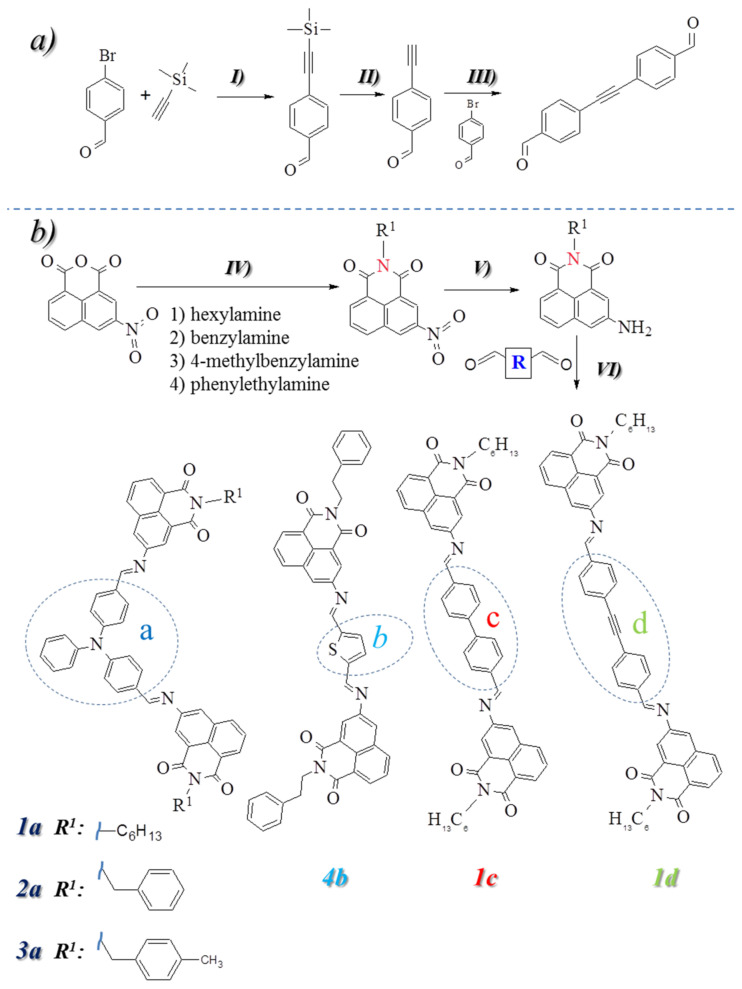
(**a**) Synthesis of dialdehyde: (I) 4-bromobenzaldehyde, trimethylsilylacetylene, Et_3_N, PdCl_2_, PPh_3_,Cu, 65 °C, 3 h, (II) MeOH, K_2_CO_3_, 2 h, rt., (III) 4-bromobenzaldehyde, 4-ethynylbenzaldehyde, Pd(PPh_3_)_2_Cl_2_, PPh_3_, CuI, Et_3_N, 70 °C, 15 h. (**b**) Synthesis of the compounds: (IV) 3-nitro-1,8-naphthalic anhydride, amine (1, 2, 3 or 4), EtOH, 2 h in reflux, (V) 10% Pd/C, EtOH, hydrazine, 60 °C, N_2_, 6 h, (VI)EtOH, CF_3_COOH, dialdehyde (a, b, c, d).

The bis-(imino-1,8-naphthalimides) were obtained in a three-step reaction (Figure 1b). In the first stage, the condensation of commercially available 3-nitro-1,8-naphthalic anhydride with various amines, such as (1) hexylamine, (2) benzylamine, (3) 4-methylbenzylamine, (4) 2-phenethylamine in the ethanol was performed. Next, the nitro group was reduced to the amine by using 10% Pd/C as catalyst and hydrazine in ethanol [46,57]. Then, a reaction was carried out between the synthesized amines and commercially available dialdehydes such as: (a) 4,4′-diformyltriphenylamine, (b) 2,5-diformylthiophene, (c) 4,4′-biphenyldicarbaldehyde, as well as (d) dialdehyde obtained in the Sonogashira reaction. The obtained targeted compounds are presented in Figure 1.

The chemical structure and purity of synthesized final compounds were confirmed based on ^1^H NMR, ^13^C NMR (Figures S2 and S3 in ESI), and elemental analysis. Moreover, for molecule 1a the correlation spectra COSY and HMQC have performed for more detailed analysis of its structure (Figure S4 in ESI). In HMQC spectrum seventeen carbon atoms correlated with hydrogen atoms are seen (Figure S4). Two signals of isolated carbons in the imide part in the range of 160 ÷ 164 ppm are observed (Table 1). Thus, based on the analysis of the 2D spectra, signals for the imine bond in the other compounds were assigned (Table 1).

The chemical shift for the carbons in the imine bond depends on the core structure (a, b, c, and d presented in Figure 1). Compounds containing an aryl core (c and d) have a carbon assigned to imine (–N=CH–) about 150 ppm, with thiophene core at 154 ppm (b) and with triphenylamine (TPA) core at 161 ppm (a). In comparison, the signals of the imine in the range of 8.6–8.9 ppm in the ^1^HNMR spectrum were seen (Table 1). Experimental content of nitrogen, carbon, and hydrogen atoms were found to be consistent with the theoretical value.

### 3.2. Thermal Characterization

**Figure 2:**
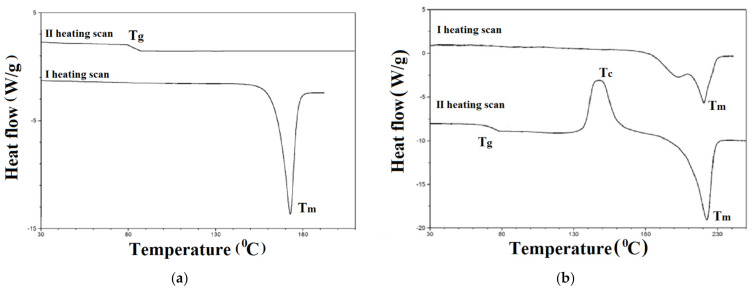
The DSC thermograms of (**a**) 1a and (**b**) 1c registered in I and II heating scan.

The thermal stability, phase transition temperatures (T_m_, T_c_), and glass transition temperatures (T_g_) of bis-(imino-1,8-naphthalimide) derivatives were determined by the thermogravimetric analysis (TGA) and the differential scanning calorimetry (DSC), respectively. Organic materials dedicated to electronic applications should show a sufficiently high temperature of the beginning of thermal decomposition (T_5_), and melting (T_m_) or glass transition temperatures (T_g_), which allows for trouble-free integration in devices [46,58,59,60]. The collected data from thermal investigations are presented in Table 2. The exemplary DSC thermograms are given in Figure 2. (for other compounds in Figure S6).

The presented bis-(imino-1,8-naphthalimide) derivatives exhibited temperatures of 5% weight loss above 280 °C. Considering the impact of core structure comparing 1a, 1c and 1d the highest T_5_ = 426 °C was recorded for the compound with triphenylamine core and hexyl chain (1a). The compounds with triphenylamine (1a–3a) showed different T_5_ dependent on the structure attached to the nitrogen in the imide ring. The T_5_ was grown from 426 °C (1a) with hexyl chain to 431 °C (2a) with benzyl and 446 °C (3a) with 4-methylbenzyl (1a < 2a < 3a). This same behavior, growing T_5_ dependent on the substituent attached to the nitrogen atom in imide unit, was noticed in our previous publication [61]. The temperature of 5% weight loss was lowered by the presence of the ethynyl bond (1d) compared to compound 1c, moreover the two steps of decomposition of 1d was noticed. The first step of decomposition of 1d at the 275 °C can be assigned to degradation of ethynyl linkage [62].

The melting temperature (T_m_) as the endothermic peak was registered during the first heating scan in the range of 173–259 °C, which indicates that bis-(imino-1,8-naphthalimide) derivatives were obtained as crystalline compounds [46]. During the second heating scan (after rapid cooling) the glass transition temperature (T_g_) was recorded in the range 74–140 °C (Table 2). In the case of compounds with thiophene (4b) and biphenyl (1c) structure upon further heating above T_g_, the “cold crystallization temperature” (T_c_, as the exothermic peak) and T_m_ were seen (Figure 2b). The presence of the glass transition temperature confirmed the ability of investigated molecules to transform from crystalline into the amorphous state. Thus, bis-(imino-1,8-naphthalimide) derivatives are molecular glasses [45,63]. The lack of tendency for crystallization in the second heating scan was confirmed for 1a–3a and 1d, which means that the presented molecules are stable molecular glasses. The T_g_ was strongly depended on the chemical structure where the highest temperature was recorded for the compounds with TPA core and benzyl ring (2a) and the lowest for 1c with biphenyl and hexyl chain (T_g_ = 74 °C). 

Symmetrical 1,8-naphthalimide with TPA core and hexyl chain (1a) had higher glass transition temperature (T_g_ = 86 °C) than its unsymmetrical analogue—3-(4-(diphenylamine)-N-benzo)-N-hexyl-1,8-naphthalimide (T_g_ = 51 °C) described in our former work [46]. This same tendency was noticed for T_5_ and T_m_. In the case of symmetrical 1,8-naphthalimide with thiophene core and phenylethyl structure (4b) the higher T_m_ and T_g_ were registered compare with symmetrical imide with thiophene core and hexyl chain—5,5′-(thiophene-2,5-diylbis(methan-1-yl-1-yli-dene))bis(azan-1-yl-1-ylidene)bis(2-hexyl-1H-benzo[de]isoquinoline-1,3(2H)-dione), (T_m_ = 239 °C; T_g_= 84 °C) described in our former publication [45].

### 3.3. Electrochemical Investigations

**Figure 3:**
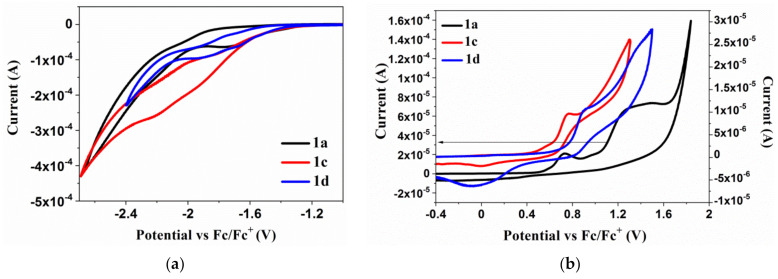
The voltammograms of the (**a**) reduction and (**b**) oxidation processes of the 1a, 1c, 1d (Pt, v = 0.1V/s, 0.1 mol/dm^3^ Bu_4_NPF_6_ in CH_2_Cl_2_ with 10^−3^ mol/dm^3^ of compounds).

Electrochemical investigations were performed by cyclic voltammetry (CV) and differential pulse voltammetry (DPV) in 0.1M Bu_4_NPF_6_ electrolyte in dichloromethane (DCM) (10^−3^ mol/dm^3^). Based on the CV and DPV voltammograms the onset potentials of reduction, and oxidation (E_red(onset)_ and E_ox(onset)_) were determined and the electron affinities (EA) and ionization potentials (IP) were estimated. The cyclic voltammograms of the 1a, 1c, 1d compounds are presented in Figure 3. whereas, for others are shown in Figure S7. The electrochemical data are collected in Table 3. 

The ionization potentials (IP) were obtained between −5.80 and −5.46 eV, and the electron affinities (EA) between −3.91 and −3.22 eV with the energy band gap (E_g_) below 2.39 eV (above 1.66 eV). The obtained derivatives show the irreversible oxidation processes as well as irreversible reduction processes, except for 2a (with triphenylamine and benzyl), 3a (with triphenylamine and 4-methylbenzyl) and 4b (with thiophene and phenylethyl), where the quasi-reversible first reduction process was seen (ΔE = 80 mV for 3a and ΔE = 110 mV for 2a, ΔE = 110 mV for 4b) (see Figure S7). The reduction process is related to acceptor moieties namely to the azomethine linkage (–N=CH–) and imide ring where the reduction process can occur. The first reduction step was seen between −1.46 and −1.99 V, dependent on the structures of the compounds, and indicates the reduction of –N=CH– [45]. 

The reduction process C=O → C=O^−^ in the imide ring was seen as two peaks (E_red_^2^ and E_red_^3^). The properly separated and defined peaks were registered by the DPV method except for compound 1c, where the one broad peak was shown (also during the oxidation process). Presence of the ethyne-1,2-diylbenzene structure strongly affected on the E_red_^1^ position which is seen comparing the compound 1c (E_red_^1CV^ = −1.92 V) and 1d (E_red_^1CV^ = −1.66 V). The synthesized bis-(imino-1,8-naphthalimide) derivatives were characterized by the multi-step processes of oxidation, except for 1c. The first oxidation process, seen about 0.65 V, can be assigned to the oxidation of the imine bond and may vary depending on the donor substituent to the –N=CH– bond. The other E_ox_ peaks are connected with donor moieties attached to the imine bond and the nitrogen in the imide ring as was reported in our previous work [46].

The unsymmetrical analog of 1a, reported in our former work [46], exhibited both, oxidation and reduction, irreversible processes. The presence of the second 1,8-naphthalimide group lowered the E_red_ and E_g_. However, as was mentioned earlier, the quasi-reversible first reduction process for compounds with triphenylamine core (2a and 3a) was observed, which means that presence of benzyl ring (2a) and 4-methylbenzyl (3a) strongly affected the first reduction process. Differences were also notice for IP, EA and E_g_ values, between 5,5′-(thiophene-2,5-diylbis(methan-1-yl-1-yli-dene))bis(azan-1-yl-1-ylidene)bis(2-hexyl-1H-benzo[de]isoquinoline-1,3(2H)-dione) and 5,5′-(thiophene-2,5-diylbis(methan-1-yl-1-yli-dene))bis(azan-1-yl-1-ylidene)bis(2-(3,5-dimethylphenyl)-1H-benzo[de]isoquinoline-1,3(2H)-dione), described in our former publication [45] and compound 4b, presented in this work. Ionization potentials and electron affinities levels are higher for 4b molecule (with phenylethyl substituent) than for other compounds. 

It can be noticed the value of the energy gap in the tested compounds (1a–3a, 4b, 1c, 1d) are relatively lower (1.58–2.36 eV) compared to the compounds 1–20, collected in Table S1 (E_g_ > 2.10 eV). Furthermore, based on the cyclic voltammetry studies in CH_2_Cl_2_, it can be concluded that the presence of an azo- or imino-linker (2.10–2.68 eV) seems to be important for lowering energy gap in contrast to the derivatives with C–C bond (e.g., -C≡C-, E_g_ = 2.55–2.94 eV). This dependence may be due to better electrochemical activity of azo- and imine- derivatives in contrast to other analyzed compounds (Figure S1, Table S1). In this case, the indication of the linker seems to be justified, because the above-mentioned tendency can be described independently of the core and the substituent in the imides part for analyzed compounds (Figure S1, compare molecules 3–6 with 9–14). 

### 3.4. Theoretical Calculations

The theoretical calculations were performed with the density functional theory (DFT) carried out using the Gaussian09 program on B3PW91/6-311g^++^ level. The molecular geometry of the singlet ground and first excited states of the compounds were optimized in the gas phase and its electronic structures and electronic transitions were calculated with use of the Polarizable Continuum Model (PCM) in dichloromethane for comparison of HOMO, LUMO energies with electrochemical data and in chloroform. The optimized geometries of the compounds are depicted in Figure S8. in the ESI. In the ground state molecules of the compounds display deviation from planarity within the central part (TPA (a series), thiophene (4b), biphenyl (1c) and ethyne-1,2-diylbenzene (1d)) and 1,8-naphthalimide (Table S2). Comparing the energies of HOMOs and LUMOs determined on the basis of electrochemical data (Table S6) with theoretically calculated values it can be noticed that the calculated HOMO energies of a series (TPA core) compounds correspond with the experimental values of IP determined from CV measurements. The discrepancies between the experimental and the calculated energy values of the HOMO level for 1c, 1d and 4b are greater. The calculated LUMOs energies were overestimated but the calculated values of the HOMO and LUMO energies were used only for consistency with geometry optimization. For a more detailed description of the molecular orbitals the contribution of a molecule parts, i.e., core, –N=CH–, 1,8-naphthalimide and substituent R to a molecular orbital was calculated. The obtained DOS diagrams are presented in Figure S10. in the ESI and composition of selected molecular orbitals are gathered in Table S3. Calculations show that LUMO in the compounds of 1a–3a and 1c is localized on the 1,8-naphthalimide part. In 1d and 4b LUMO comprises the central molecule part with the azomethine–1,8-naphthalimide fragment. HOMO is localized on the triphenylamine core of the 1a–3a compounds. In the case of 1c, 1d and 4b molecules HOMO comprises the central part (biphenyl (1c), ethyne-1,2-diylbenzene (1d), thiophene (4b)) including 1,8-naphthalimide linked by imine bond. The aromatic substituents on the 1,8-naphtalenediimide nitrogen play role at the HOMO-3, HOMO–4 levels, in the case of 2a, 3a, and especially in 4b compound where HOMO-2 is localized on the phenylethyl substituent (Table S3). The transition corresponding to the excitation wavelength (340 nm vide infra) has, in all compounds, a mixed intra molecular charge transfer/locally excited (ICT/LE) nature (Table S4). However, in the case of 2a, the excitation is related to the charge transfer from 1,8-naphthalimide to triphenylamine core, and in 3a from TPA to the 1,8-naphthalimide fragment with imine linker. The R substituents take part in the excitation process in the case of compound 4b for which the transition is related to π → π* transition.

### 3.5. Photophysical Properties 

**Figure 4:**
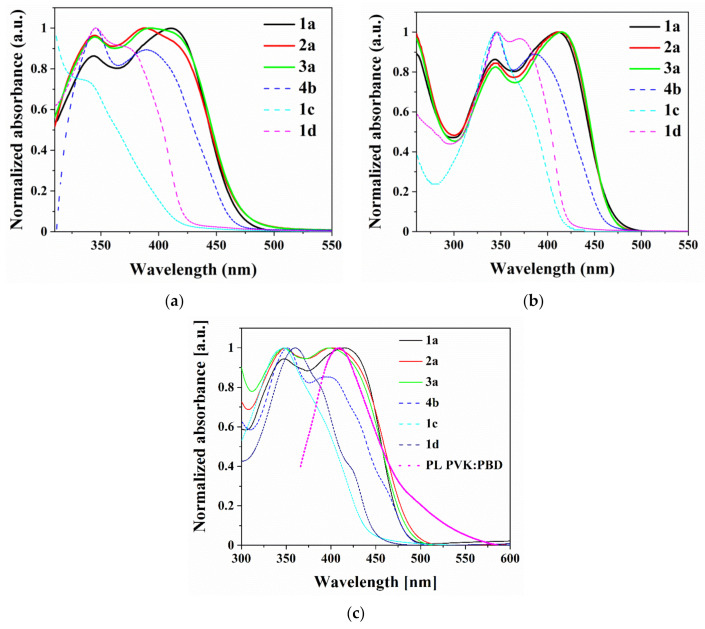
Absorption spectra of bis-(imino-1,8-naphthalimide) derivatives in (**a**) N-methyl-2-pyrrolidone; (**b**) chloroform, and (**c**) film together with PL spectrum of PVK:PBD.

The electronic absorption (UV-Vis) and photoluminescence (PL) spectra were recorded in non-polar and polar solvents, such as chloroform and N-methyl-2-pyrrolidone (NMP) (c= 10^−5^ mol/dm^3^), and in the solid-state as films and blends with PVK:PBD matrix on the glass substrates. The UV-Vis spectra are presented in Figure 4. Data from absorption and emission measurements are collected in Table 4.

Bis-(imino-1,8-naphthalimide) in chloroform and NMP solutions were absorbed electromagnetic radiation with λ_max_ in the range 243–415 nm (5.10–2.99 eV). The absorption bands in the higher energy (λ_max_ = 243–274 nm) can be attributed to π → π* electron transitions in the aromatic rings, while the λ_max_ above 328 nm to π →π* naphthalimide [46,64]. The λ_max_ in the lower energies, above 360 nm, belong to the charge transfer (CT) between the core (at the 3-C position) and naphthalimide [64]. The shift of the maximum of the absorption band in CHCl_3_ and NMP towards lower energies was observed for bis-(imino-1,8-naphthalimides) with a triphenylamine (TPA) core (1a–3a) compared to other compounds (Figure 4). The shift of λ_max_ towards lower energies indicates a better degree of conjugation for 1a–3a, confirmed by NMR measurements.

The same position of λ_max_ in chloroform and N-methyl-2-pyrrolidone for the compound 1d with ethyne-1,2-diylbenzene substituent was observed, suggesting that the difference in dipole moments between the excited and the ground state are minimal [40]. The bathochromic shift of λ_max_ of molecule with a triple bond (-C≡C-), (1d) compared to compound with the biphenyl core (1c) in solutions were noticed (∆λ_max_ = 35 nm) (Table 4). No differences in λ_max_ position between molecules with TPA core in chloroform solution were seen, however, dissimilarities were recorded in emission spectra. The hypsochromic shift of λ_max_ of molecules with benzyl (2a, λ_max_ = 387 nm) and 4-methylbenzyl (3a, λ_max_ = 397 nm) attached to the nitrogen in the imide ring compared to 1a with hexyl chain (λ_max_ = 413 nm) registered in NMP was seen.

Bis-(imino-1,8-naphthalimide) derivatives absorbed radiation in films with λ_max_ in the range 345–413 nm. The hypsochromic shift in films was noticed for molecules with TPA core depending on the substituent in the imide unit (hexyl chain 1a, λ_max_= 413nm > phenyl ring 2a, λ_max_ = 402 nm > 4-methylphenyl 3a, λ_max_ = 397 nm). Thus, comparing film with chloroform solution, the hypsochromic shift of ∆λ_max_= 13 nm (2a) and ∆λ_max_ = 18 nm (3a) was seen. Differences in the λ_max_ for other molecules were slight (∆λ_max_ = 2–7 nm).

The second naphthalimide group, present in 1a molecule, allowed to obtain bathochromically shifted absorption spectrum (by approximately 31 nm in CHCl_3_ and 36 nm in the film) comparing to molecule presented in our former work [46] 3-(4-(diphenylamine)-N-benzo)-N-hexyl-1,8-naphthalimide, due to difference in the degree conjugation. The position of the maximum absorption bands for 5,5′-(thiophene-2,5-diylbis(methan-1-yl-1-yli-dene))bis(azan-1-yl-1-ylidene)bis(2-hexyl-1H-benzo[de]isoquinoline-1,3(2H)-dione) and 5,5′-(thiophene-2,5-diylbis(methan-1-yl-1-yli-dene))bis(azan-1-yl-1-ylidene)bis(2-(3,5-dimethylphenyl)-1H-benzo[de]isoquinoline-1,3(2H)-dione) (published in former work [45]) and 5,5′-(thiophene-2,5-diimine)-bis(2-(2-phenethyl)-1H-benzo[de]isoquinoline-1,3(2H)-dione) (4b) are very similar, which allows concluding that interactions between thiophene core-imine bond and imide unit are more crucial compare to substituent in the imide ring.

**Figure 5:**
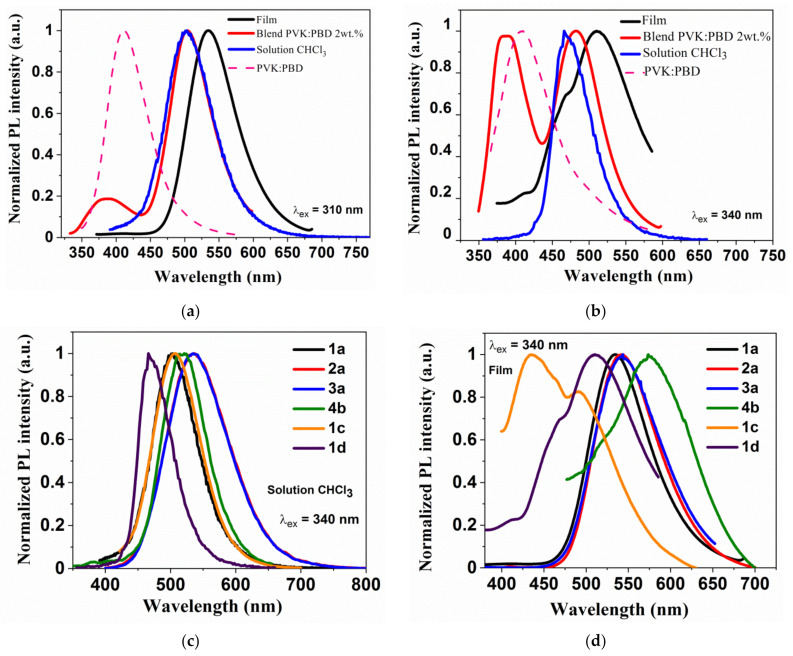
Emission spectra (PL) (**a**) of 1a; (**b**) 1d; (**c**) in chloroform solution and in (**d**) the films of bis-(imino-1,8-naphthalimide) derivatives.

Bis-(imino-1,8-naphthalimides) showed the ability to emission of light in solutions. In PL spectra one band with maximum (λ_em_) in the range of 450–544 nm from blue to green light was observed (Figure 5c). The emission data are summarized in Table 4. The naphthalimide 4b with thiophene core and 1c with biphenyl core showed the λ_em_ in the blue spectral region in both solutions. The red shift of λ_em_ in NMP was seen for 1a and 1d compared to CHCl_3_ (blue spectral region in CHCl_3_, green spectral region in NMP, see Figure S12) under external UV-light λ_ex_= 366 nm, but for 2a and 3a, the opposite behavior was noticed. The various excitation wavelengths (λ_ex_) did not affect the maximum PL band (λ_em_) in accordance with the Kasha’s rule.

The photoluminescence spectrum of the molecule with triphenylamine core and hexyl chain (1a) was hypsochromically shifted about 32 nm in CHCl_3_ solution compared to PL spectrum of compounds with benzyl (2a) and 4-methylphenyl (3a) (Table 4). An opposite behavior was detected in the NMP solution. Furthermore, in the solid-state in the films and blends, this behavior was not seen (λ_em_ at about 540 and 500 nm). The quantum yield (Φ_PL_) in the films of 1a–3a was about 4%, but compounds in chloroform showed higher Φ_PL_ 25, 26 and 17% for 1a, 2a, 3a, respectively. In the S_1_ state the planarity distortion of the 1,8–naphthalimide–X–1,8–naphthalimide part is reduced, which has impact on the delocalization and conjugation of π-electrons in the molecules. Changing the molecule’s geometry in the excited state is easier in the solution than in film which may explain the very low quantum yields of the film emissions. The most bathochromically shifted spectrum was recorded for the molecule with thiophene core and phenylethyl (4b) in film (λ_em_ = 577 nm, the yellow spectral region; Figure 5d) with the lowest Φ_PL_ (also in CHCl_3_, Φ_PL_ = 1%). The PL maxima for all the molecules in the films were bathochromically shifted compared to the chloroform solution, except for 1c (Figure S14).

In the next step of the research the PL spectra of the poly(N-vinylcarbazole) (PVK):2-tert-butylphenyl-5-biphenyl-1,3,4-oxadiazole (PBD) (1:1) blends were registered, where the energy transfer can take place (Förster transfer or exchange (Dexter) mechanism) [65,66]. The energy transfer process in the host-guest structure may occur, consisting of the energy transfer from the host matrix (PVK:PBD) to the guest molecule in the ground state [59]. The above mechanisms are processes of non-radiative energy transfer and may coexist in the case of minimal distances between the guest and the host. The Förster Energy Transfer (FRET) results from dipole-dipole interactions and the Dexter energy transfer requires overlapping of electron clouds [59]. Effective energy transfer occurs when the emission intensity of the guest increases and the host decreases in the host’s presence [67,68]. Furthermore, the effective FRET energy transfer is possible when host’s emission is overlapping with the absorption spectrum of the guest [65,68]. The PL spectrum of the matrix (PVK:PBD) overlapping with the absorption spectrum in the case of the molecules with TPA (1a–3a) and thiophene (4b) core, for the other molecules partial overlap was seen (Figure 4c). In PL spectra of the blends, one (for 4b) or two bands were found (Figure S14). The band with λ_em_ about 390 nm came from the emission of the PVK:PBD matrix and the other originate from the bis-(imino-1,8-naphthalimides) emission (see Table 4, Figure 5a,b). The presence of the second emission band (at about 390nm) in the PL spectra of PVK:PBD blends indicates no complete energy transfer. It is worth mentioning that for compound 1a, with TPA and hexyl chain, the highest value of the quantum yield in the PVK:PBD blends was obtained (Φ_PL_ = 11.3%).

The unsymmetrical analogue of 1a with TPA core, presented in our previous work [46] emitted light with the same λ_em_ position in the solid-state but in solutions, the red shift (in CHCl_3_,∆λ_em_ = 64 nm) and blue shift (in NMP, ∆λ_em_ = 32 nm) was observed. The unsymmetrical 3-(4-(diphenylamine)-N-benzo)-N-hexyl-1,8-naphthalimideexhibited lower Φ_PL_ and shorter lifetime (τ) compared to 1a [46]. The PL spectrum in CHCl_3_ of the molecule 4b is bathochromically shifted compared to the compound with the 3,5-dimethylphenyl substituent (∆λ_em_ = 18 nm) presented in publication [46]. Moreover, the compound 2a showed hypsochromic shift of the UV-Vis and PL spectra (∆λ_max_ = 16 nmin CHCl_3_ and 33 nm in film; ∆λ_em_ = 47 nm in CHCl_3_ and 7 nm in film) compared to the synthesized 1,8-naphthalimides with TPA at 4-C position (C–C linker) and benzyl ring in the imide unit (N-C bond) presented by Arunchai et al. [40]. Differences were also noticed in Φ_PL_ and τ, where 5,5′-(triphenylamine-4,4′-diimine)-bis(2-(2-benzyl)-1H-benzo[de]isoquinoline-1,3(2H)-dionehad the longest life-time but lowest Φ_PL_ in the chloroform solution compared to naphthalimide [65].

The most similar values of the λ_max,_ λ_em_ and quantum yield are represented by bisimides with carbon–carbon aryls linkers (at 4-C position of thiophene (18), EDOT(19) and bithiophene(20), Table S1) compared to the compounds presented in this work. Moreover, the collected molecules in the Table S1 (ESI) and bis-(imino-1,8-naphthalimides) emitted light from blue to green spectral region. 

### 3.6. Electroluminescence Study

**Figure 6:**
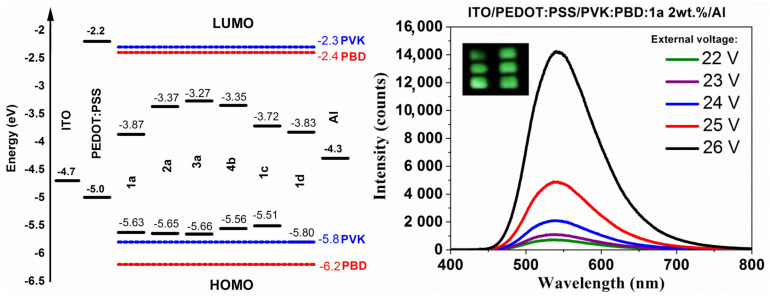

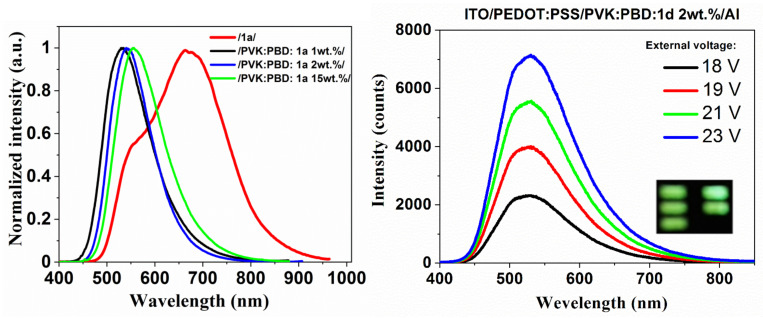
Diagram of HOMO and LUMO energy levels of diode components and EL spectra of the working devices under an applied voltage with their photo.

The synthesized bis-(imino-1,8-naphthalimides) were used as an active layer or as components of the active layer in the organic electroluminescence diodes (OLEDs). The following configuration of devices ITO/PEDOT: PSS/molecules/Al and ITO/PEDOT: PSS/PVK: PBD: molecules 2 wt.%/Al were applied. PVK is often used in organic electronics to build an active layer due to the high mobility of holes [59,69]. PVK was mixed with PBD, which exhibited high mobility of electrons, creating a two-component matrix and finally a host-guest active layer [66,70,71]. Data from electroluminescence (EL) measurements are collected in Table 5 and the EL spectra of chosen fabricated devices are presented in Figure 6 (and Figure S16).

Only two devices, which the bis-(imino-1,8-naphthalimides) acts as an active layer, with the molecules 4b and 1d, did not show electroluminescence, for other diodes the red light was registered (λ_EL_ ≈ 675 nm). A lack of emission induced by external voltage of devices with compound 4b (thiophene core and phenylethyl substituent) can be explained by low Φ_PL_ and short PL lifetime (τ) in the solid state. The presence of 2 wt.% compounds dispersed molecularly in the matrix as active layer in diode allowed for registered emission of light with the maximum EL band (λ_EL_) at the green visible spectral region (Figure 6).

The highest electroluminescence intensity was registered for device with triphenylamine core and hexyl chain (1a) dispersed in PVK:PBD matrix (2 wt.% content of 1a) (Table 5). Due to the high EL intensity the device with PVK:PBD contains 1 and 15 wt.% of 1a were prepared (Figure 6). It was showed that 15 wt.% content of the 1a molecule in the PVK:PBD matrix allowed to obtain the diode with the highest electroluminescence intensity, however the external voltage was high. It should be emphasized that the EL spectra were registered under external voltage about 10 V. The guest-host devices with 1a emitted green light with the shift of λ_EL_ position together within crease of its content in the PVK:PBD matrix (λ_EL_ = 530 nm < 540 nm < 555 nm). 

The energy levels of the HOMO and LUMO orbital’s of bis-(imino-1,8-naphthalimides) are respectively higher and lower than a binary matrix energy levels (Figure 6), which may indicate the dominance of the trapping mechanism [68,72]. However, in the recombination process in OLEDs, the processes of energy transfer (FRET or/and Dexter) and the charge trapping mechanism may coexist [73]. Comparing EL spectra with PL spectra the λ_EL_ shift towards longer wavelengths was observed (Table 4 and Table 5). This behavior can be attributed to electroplex formation, both in the bis-(imino-1,8-naphthalimides), which can also explain shoulder in the case of device with an active layer containing 1a molecule, as well as in the guest-host structures [49,74]. At this stage of research, the diode parameters (luminance, luminous efficacy) were not measured. 

The device based on the unsymmetrical 3-(4-(diphenylamine)-N-benzo)-N-hexyl-1,8-naphthalimide (described in [46], dispersed in PVK:PBD with 2 wt.% content) emitted light with λ_EL_ = 538 nm, which means, that the presence of an additional 1,8-naphthalimide group did not have an effect on the λ_EL_ position (compared with 1a). Larger differences were observed in the case of diodes with 15 wt.% content of molecules in the matrix (∆λ_EL_ = 14 nm).

## 4. Conclusions

Six symmetrical bis-(imino-1,8-naphthalimides) with triphenylamine (TPA), thiophene, biphenyl, and ethyne-1,2-diylbenzenecoreinthe 3-C position of naphthalene structure and hexylamine (1a,1c,1d), benzylamine (2a), 4-methylbenzylamine (3a), and 2-phenethylamine (4b) substituents in the imide ring were synthesized. These molecules showed temperature of 5% weight loss above 280 °C and were obtained as crystalline compounds with ability to transform into stable molecular materials. The glass transition temperature was strongly dependent on the chemical structure. Bis-(imino-1,8-naphthalimides) were electrochemically active and showed the energy band gap below 2.39 eV. Based on the DFT calculations, HOMO is localized on the central part of the compounds—core, and in the case of 1c, 1d, and 4b is mixed (core–imine bond–naphthalimide). LUMO is localized on the 1,8-naphthalimide part, and for 1d and 4b LUMO is also mixed. The presented molecules emitted blue, green and yellow light in the investigated media with the higher quantum yields in solutions (except 1d and 4b). The bis-(imino-1,8-naphthalimides) were acting as the active layer and as a component of the active layer in the preliminary EL studies. The highest EL intensity was registered for the device with the 15 wt.% of 1a (triphenylamine core and hexyl chain) in PVK:PBD matrix. Molecule 1a, in our opinion, is promising for optoelectronic applications and can be further investigated and modified as a component of the binary blends or green/red light emitter. 

## Figures and Tables

**Table 1:** Selected NMR chemical shifts (ppm) and multiplicities for compounds.

Code	Imide ^13^C (ppm)	Imine	
		^1^H (ppm)	^13^C (ppm)
1a	164.1, 164.0	8.6 (s, 2H)	161.2
2a	-	8.6 (s, 2H)	-
3a	164.3, 165.1	8.6 (s, 2H)	161.2
4b	163.9, 164.8	8.9 (s, 2H)	154.1
1c	164.0, 161.5	8.7 (s, 2H)	150.3
1d	164.4, 160.9	8.7 (s, 2H)	149.9

**Table 2:** Thermal stability and transition temperatures of investigated 1,8-naphthalimide derivatives.

Compound	TGA		DSC			
			I Heating Scan	II Heating Scan		
	T_5_^a^	T_max_^b^	T_m_^c^	T_g_^e^	T_c_^d^	T_m_^c^
	(°C)	(°C)	(°C)	(°C)	(°C)	(°C)
1a	426	464	173	86	nd	nd
2a	431	479	259	140	nd	nd
3a	446	488	209	138	nd	nd
4b	428	498	255	118	170	226
1c	387	432	220	74	148	225
1d	285	275, 479	251	127	nd	nd

^a^ T_5_, T_10_—temperature based on 5 and 10% weight loss from TGA curves, ^b^ Temperature of the maximum decomposition rate from DTG curves. ^c^ Melting temperature, ^d^ Cold crystallization temperature, ^e^ Glass transition temperature, nd—not detected.

**Table 3:** Electrochemical data of the bis-(imino-1,8-naphthalimide) derivatives.

Compound	Method	E_red_^1^	E_red(onset)_	E_ox_^1^	E_ox(onset)_	EA	IP	E_g_
		[V]	[V]	[V]	[V]	[eV]	[eV]	[eV]
1a	CV	−1.72 ^a^	−1.23	0.73 ^a^	0.53	−3.87	−5.63	1.76
	DPV	−1.46	−1.22	0.50	0.36	−3.88	−5.46	1.58
2a	CV	−1.82 ^b^	−1.73	0.69 ^a^	0.55	−3.37	−5.65	2.28
	DPV	−1.89	−1.77	0.61	0.53	−3.33	−5.63	2.30
3a	CV	−1.96 ^b^	−1.83	0.67 ^a^	0.56	−3.27	−5.66	2.39
	DPV	−1.99	−1.88	0.58	0.48	−3.22	−5.58	2.36
4b	CV	−1.75 ^b^	−1.53	0.76 ^a^	0.46	−3.35	−5.56	2.21
	DPV	−1.67	−1.57	0.78	0.56	−3.43	−5.66	2.23
1c	CV	−1.92 ^a^	−1.38	0.76 ^a^	0.41	−3.72	−5.51	1.79
	DPV	−1.96	−1.28	0.70	0.38	−3.82	−5.48	1.66
1d	CV	−1.66 ^a^	−1.27	0.90 ^a^	0.70	−3.83	−5.80	1.97
	DPV	−1.62	−1.19	0.80	0.70	−3.91	−5.80	1.89

IP = −5,1-E_ox(onset)_·|e|, EA = −5,1-E_red(onset)_·|e|, E_g_ = E_ox(onset)_-E_red(onset)_. Measurements inCH_2_Cl_2_ with concentration 10^−3^ mol/dm^3^ and electrolyte 0.1 mol/dm^3^Bu_4_NPF_6_. Pt as the working electrode. ^a^ Irreversible process. ^b^ Quasi-reversible process. Ered ^1^–the first reduction process, Eox ^1^–the first oxidation process. Ered(onset)–the onset potential of the first reduction process, Eox(onset)–the onset potential of the first oxidation process.

**Table 4:** Collected UV-Vis and PL properties of bis-(imino-1,8-naphthalimides).

Compound	Medium	UV-vis	PL				
		λ_max_ (nm) (ε·10^4^) ^a^	λ_em_(nm)	Stokes Shift	Φ (%)	τ ^c^ (ns)	X^2^
				(cm^−1^) ^b^			
1a	CHCl_3_^d^	345^(2.5)^, 411^(9.8)^	501	6356	25	16.9	1.045
	NMP ^d^	342^(2.5)^, 413^(2.8)^	544	5831	-	-	-
	FILM	345, 413	537	5591	3.8	12.5	0.930
	PVK:PBD ^e^	310 ^sh^, 344 ^sh^, 424	388, 504	-	11.3	-	-
2a	CHCl_3_^d^	246^(14.1)^, 260 ^sh^, 345^(8.1)^, 415^(10.0)^	533	5335	26	19.1	1.017
	NMP ^d^	345^(3.5)^, 387^(6.9)^,414^sh^	516	6460	-	-	-
	FILM	348, 402	544	6493	4.0	16.2	1.061
	PVK:PBD ^e^	310 ^sh^, 344 ^sh^, 429 ^sh^	384, 499	-	7.5	-	-
3a	CHCl_3_^d^	246^(14.0)^, 260 ^sh^, 345^(10.1)^, 415^(12.1)^	533	5335	14	21.0	1.063
	NMP ^d^	345^(3.8)^, 393^(8.9)^, 414 ^sh^	514	6460	-	-	-
	FILM	345, 397	544	6807	3.7	15.3	1.098
	PVK:PBD ^e^	310 ^sh^, 344 ^sh^, 429 ^sh^	384, 499	-	7.3	-	-
4b	CHCl_3_^d^	246 ^sh^, 274^(23.2)^, 345^(5.8)^, 390^(4.7)^	515	6224	1	22.0	0.935
	NMP ^d^	345^(10.1)^, 387^(5.6)^	450	3618	-	-	-
	FILM	348, 397	577	4221	1.7	2.16	1.019
	PVK:PBD ^e^	310 ^sh^, 344 ^sh^	384	-	2.3	-	-
1c	CHCl_3_^d^	243^(3.6)^, 340^(9.1)^	503	9445	7	16.7	1.123
	NMP ^d^	267^(21.2)^, 340 ^(4.6)^	517	10069	-	-	-
	FILM	345	435, 490 ^sh^	5997	4.8	11.0	1.056
	PVK:PBD ^e^	310 ^sh^, 344 ^sh^	399, 477	-	3.6	-	-
1d	CHCl_3_^d^	345^(9.8)^, 376^(8.8)^	470	5319	3	11.2	1.012
	NMP ^d^	345^(17.8)^, 376^(8.8)^	525	7548	-	-	-
	FILM	360, 389 ^sh^, 424 ^sh^	471 ^sh^, 509	-	4.6	9.0	1.047
	PVK:PBD ^e^	310 ^sh^, 344 ^sh^	386, 498	-	8.4	-	-

^a^ ε—Absorption coefficient, (dm^3^·mol^−1^·cm^−1^). ^b^ Stokes shifts calculated according to the equation Δν = (1/λ_abs_ − 1/λ_em_) × 10^7^ (cm^−1^). ^c^ The average time from the multi-exponential decay profiles (Table S4). ^d^ c = 10^−5^ mol/dm^3^. ^sh^—shoulder. ^e^ 2 wt.%—concentration of compound in the matrix PVK:PBD. Underlined data indicates the dominant band.

**Table 5:** The electroluminescence data of prepared diodes.

Compound	λ_EL_	U_ELMax_	EL Intensity
	(nm)	(V)	(Counts)
1a	670 ^a^	37	2207
	530 ^b^	24	16,214
	540 ^c^	26	14,228
	555 ^d^	28	195,060
2a	675 ^a^	12	608
	535 ^c^	9	267
3a	675 ^a^	16	489
	532 ^c^	11	226
1c	675 ^a^	19	125
	525 ^c^	23	1837
1d	529 ^c^	23	7091

^a^ ITO/PEDOT:PSS/molecules/Al. ^b^ ITO/PEDOT:PSS/PVK:PBD:molecule 1 wt.%/Al. ^c^ ITO/PEDOT:PSS/PVK:PBD:molecules 2 wt.%/Al.^d^ ITO/PEDOT:PSS/PVK:PBD:molecule 15 wt.%/Al. λ_EL_—the maximum of EL band. U_ELMax_—external voltage for maximal EL intensity.

## Data Availability

Data is contained within the article or supplementary material.

## Supplementary Materials

The following are available online at https://www.mdpi.com/article/10.3390/ma14112714/s1, Figure S1: Structure of the compounds divided into the linker in the donor-acceptor system (4-C or 3-C position) (Publications: 37, 39, 40, 46, 48, 49, 50, 52), Table S1: Photophysical and electrochemical properties of 1–20 derivatives (Figure S1.) based on the literature data, Figure S2: 1HNMRof the investigated compounds (400 MHz, CDCl3), Figure S3: 13C NMR of the investigated compounds (400 MHz, CDCl3), Figure S4: HMQC and COSY correlation spectra for 1a (400 MHz, CDCl3), Figure S5: The investigated compounds under (a) 1a, (c) 1c,(d) 1d day light and (b) 1a under UV-light, Figure S6: DSC thermograms of (a) 2a, (b) 3a and (c) 4b, Figure S7: The (a) reduction and (b) oxidation processes of 2a, 3a, 4b (Pt, v= 0.1 V/s, 0.1 mol/dm3 Bu4NPF6 in CH2Cl2 with 10−3 mol/dm3 of compounds), Figure S8: Optimized geometries of the compounds (a) 1a, (b) 2a, (c) 3a, (d) 1c, (e) 1d, (f) 4b, Figure S9: Contours of HOMO and LUMO of the compounds (a) 1a, (b) 2a, (c) 3a, (d) 1c, (e) 1d, (f) 4b, Figure S10: Density-of-states diagrams of ground state of the molecules, Figure S11: Experimental and calculated UV-Vis spectra of bis-(imino-1,8-naphthalimide) derivatives, Table S2: Calculated dipole moments (in CHCl3) and geometrical parameters of the molecules in ground and first singlet excited state, Table S3: Composition of the selected MO in ground state of the compounds, Table S4: The calculated electronic transitions corresponding to excitation resulting most intense luminescence in CHCl3 solution, Figure S12: (a) 1a and (b) 1d under day light and under UV-light (λ = 366 nm) in NMP, Table S5: PL life-time (τ) measurements data, Figure S13. PL life-time (τ) curves of (a) 2a, (b) 3a and (c) 4b in chloroform solution, Figure S14. Emission spectra of (a) 2a; (b) 3a; (c) 4b and (d) 1c with PVK:PBD., Figure S15. Emission spectra of 1a and PVK:PBD, Table S6. EA, IP, HOMO, LUMO, Eg and Egopt, Figure S16. Electroluminescence (EL) spectra of the working devices under an applied voltage. Above the graphs the device structures are shown. Presented are maxima of EL intensity.
