# Supplementary material for: New Acceptor–Donor–Acceptor Systems Based on Bis-(Imino-1,8-Naphthalimide)
Source: Materials (Basel). 2021 May 21;14(11):2714. doi: 10.3390/ma14112714 (PMC8196752; doi:10.3390/ma14112714)
Supplement: Supplementary file 1 [file materials-14-02714-s001.zip › materials-1226728-supplementary.pdf]

materials

Supplementary Material

New Acceptor–Donor–Acceptor Systems Based on Bis-(Imino-1,8-Naphthalimide)

Sonia Kotowicz [1,*,†], Mateusz Korzec [1,*,†], Agnieszka Katarzyna Pająk [1], Sylwia Golba [2], Jan Grzegorz Małecki [1], Mariola Siwy [3], Justyna Grzelak [4], Sebastian Maćkowski [4] and Ewa Schab-Balcerzak [1,3]

[1] Institute of Chemistry, University of Silesia, 9 Szkolna Str., 40-006 Katowice, Poland; agpajak@us.edu.pl (A.K.P.); jan.malecki@us.edu.pl (J.G.M.); ewa.schab-balcerzak@us.edu.pl (E.S.-B.)
[2] Institute of Materials Engineering, University of Silesia, 75 Pulku Piechoty Str., 41-500 Chorzow, Poland; sylwia.golba@us.edu.pl
[3] Centre of Polymer and Carbon Materials, Polish Academy of Sciences, 34 M. Curie-Sklodowska Str., 41-819 Zabrze, Poland; msiwy@cmpw-pan.edu.pl (M.S.); ebalcerzak@cmpw-pan.edu.pl (E.S.-B.)
[4] Faculty of Physics, Institute of Physics, Astronomy and Informatics, Nicolaus Copernicus University, 5 Grudziadzka Str., 87-100 Torun, Poland; justynag@fizyka.umk.pl (J.G.); mackowski@fizyka.umk.pl (S.M.)
* Correspondence: sonia.kotowicz@us.edu.pl (S.K.); mateusz.korzec@us.edu.pl (M.K.)
† Contributed equally to this work: Sonia Kotowicz, Mateusz Korzec.

Citation: Kotowicz, S.; Korzec, M.; Pająk, A.K.; Golba, S.; Małecki, J.G.; Siwy, M.; Grzelak, J.; Maćkowski, S.; Schab-Balcerzak, E. New Acceptor—Donor—Acceptor Systems Based on Bis-(Imino-1,8-Naphthalimide). *Materials* **2021**, *14*, 2714. https://doi.org/10.3390/ma14112714

Academic Editor: Jose M. Bastidas

Received: 2 May 2021
Accepted: 18 May 2021
Published: 21 May 2021

Publisher's Note: MDPI stays neutral with regard to jurisdictional claims in published maps and institutional affiliations.

Copyright: © 2021 by the authors. Licensee MDPI, Basel, Switzerland. This article is an open access article distributed under the terms and conditions of the Creative Commons Attribution (CC BY) license (http://creativecommons.org/licenses/by/4.0/).

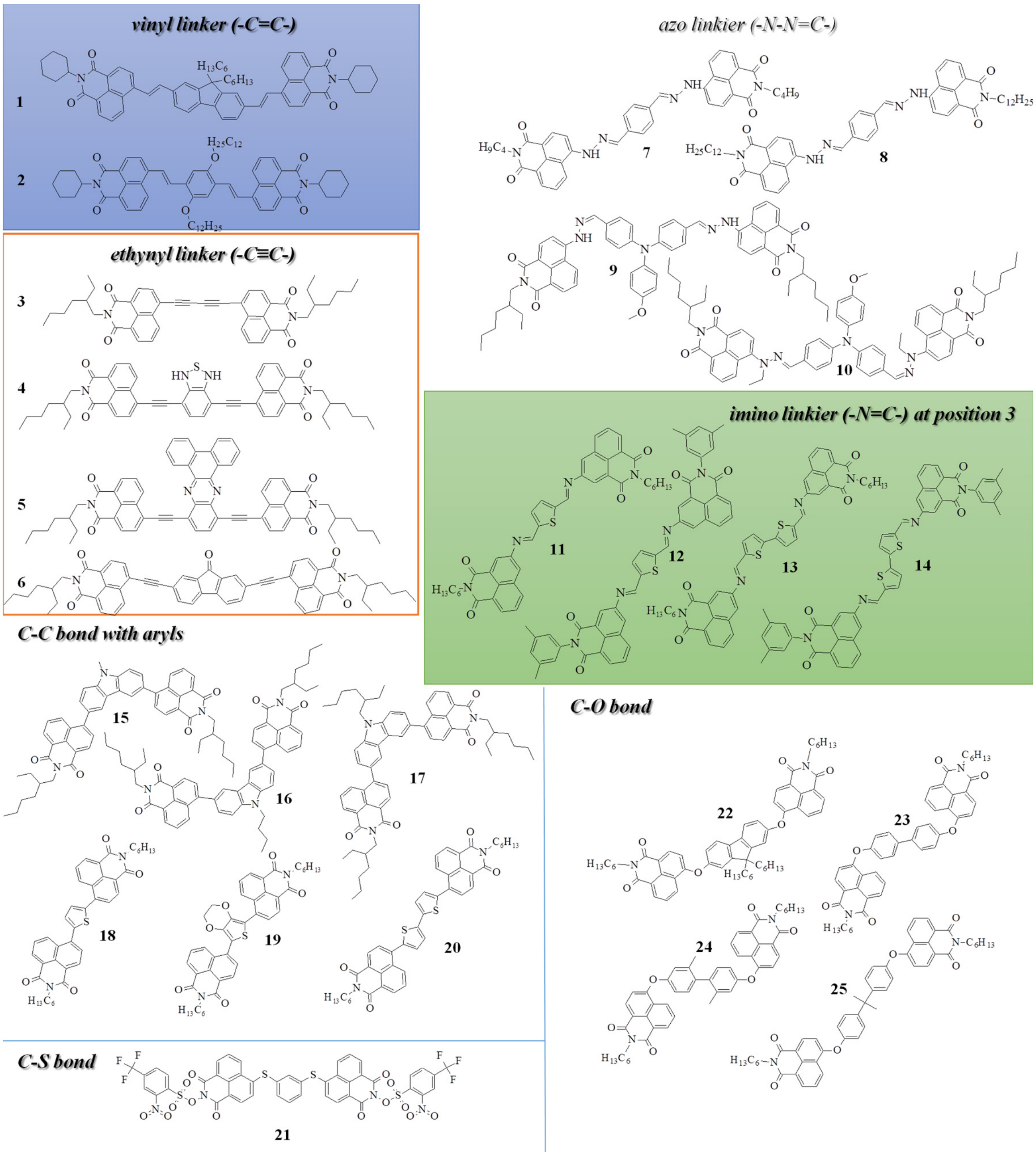

Figure S1. Structure of the compounds divided into the linker in the donor-acceptor system (4-C or 3-C position) (Publications: 37, 39, 40, 46, 48, 49, 50, 52).

Table S1. Photophysical and electrochemical properties of 1-20 derivatives (Figure S1.) based on the literature data.

No.	Medium for UV-Vis and PL	UV-Vis	PL			Medium for CV	CV			Ref.
		λ_{max} [nm]	λ_{em} [nm]	Φ	E_g^{opt} [eV]		IP [eV]	EA [eV]	E_g^{CV} [eV]	
1	THF	431	518	0.15	2.55	THF	5.37[a]	−2.99	2.55 [a]	48
2	THF	427	544	0.10	2.48	THF	5.54[a]	−2.89	2.48 [a]	
3	CH_2Cl_2	371, 392, 423	438, 464	62%	-	CH_2Cl_2	6.31	3.37	2.94	37
4	CH_2Cl_2	291, 400, 445	540	24%	-	CH_2Cl_2	5.97	3.30	2.66	
5	CH_2Cl_2	279, 329, 354, 380, 439, 463	490, 518	70%	-	CH_2Cl_2	6.03	3.48	2.55	
6	CH_2Cl_2	302, 398, 422, 447, 470	500, 526	82%	-	CH_2Cl_2	5.96	3.32	2.65	
7	Acetonitrile *	485.2	563	0.95	-	-	-	-	-	40
8	Acetonitrile *	486.3	517	0.96	-	-	-	-	-	
9	Toluene **	476	562	-	2.27	CH_2Cl_2	−5.06	−2.91	2.15	49
10	Toluene **	474	543	-	2.27	CH_2Cl_2	−5.01	−2.91	2.10	
11	Chloroform ***	244, 347, 389	499	-	-	CH_2Cl_2	−6.18	−3.50	2.68	46
12	Chloroform ***	244, 347, 388	497	-	-	CH_2Cl_2	−5.80	−3.52	2.28	
13	Chloroform ***	252, 350, 429	482	-	-	CH_2Cl_2	−5.77	−3.42	2.35	
14	Chloroform ***	244, 349, 425	504	-	-	CH_2Cl_2	−5.77	−3.46	2.31	
15	Solid films	-	541	<0.01	-	Solid	5.68	−2.94	2.74	52
16	Solid films	-	522	0.17	-	Solid	5.67	−2.95	2.72	
17	Solid films	-	522	0.06	-	Solid	5.75	−2.94	2.81	
18	Chloroform **	395	495	0.15	-	-	-	-	-	39
19	Chloroform **	416	502	0.22	-	-	-	-	-	
20	Chloroform **	428	542	0.36	-	-	-	-	-	
21	CH_2Cl_2	365	441, 533	0.001	-	-	-	-	-	50
22	CH_2Cl_2	362	437, 520	0.004	-	Acetonitrile	−6.3	−3.1	3.2	
23	CH_2Cl_2	363	428, 480	0.016	-	-	-	-	-	
24	CH_2Cl_2	363	435	0.163	-	-	-	-	-	

No. from the Figure S1. [a] Estimated either theoretically or using the optical band gap. Ref. are collected in the main publication.

* concentration = 10^{-4} M
** concentration = 10^{-5} M
*** concentration = 5 × 10-5 M.

1. NMR Spectroscopy

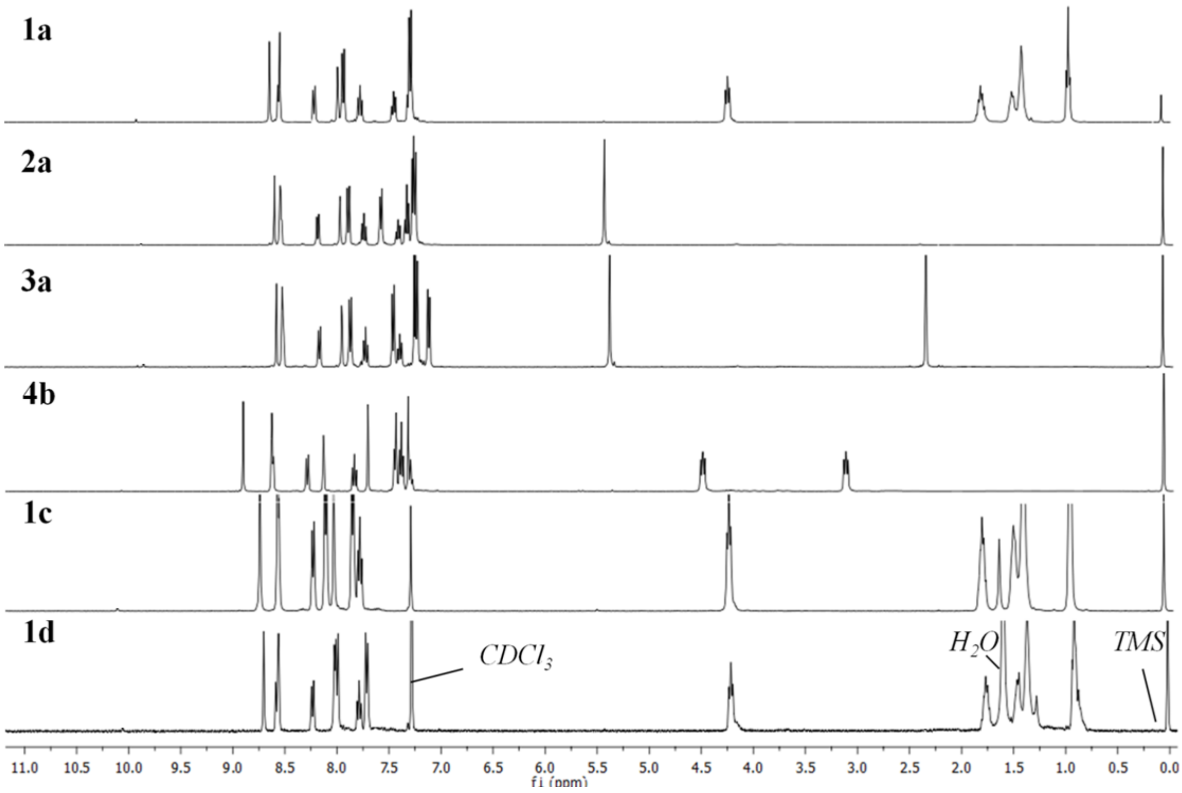

Figure S2. ^{1}HNMRof the investigated compounds (400 MHz, $CDCl_3$).

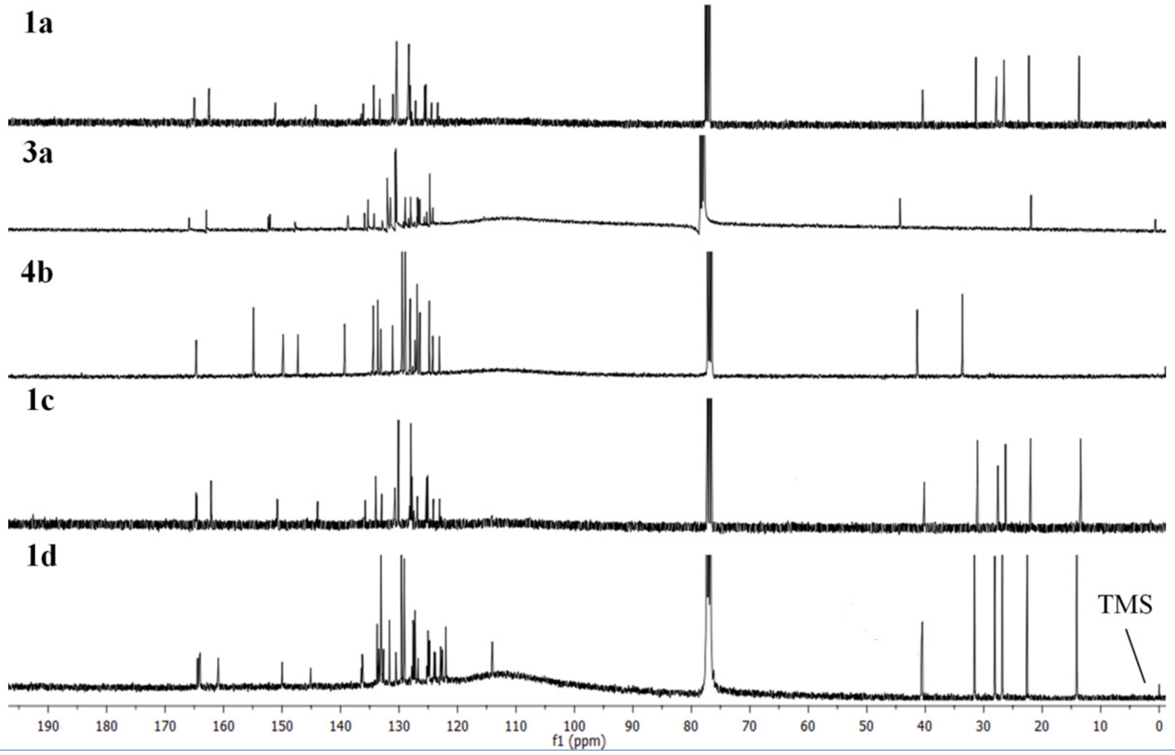

Figure S3. ^{13}C NMR of the investigated compounds (400 MHz, $CDCl_3$).

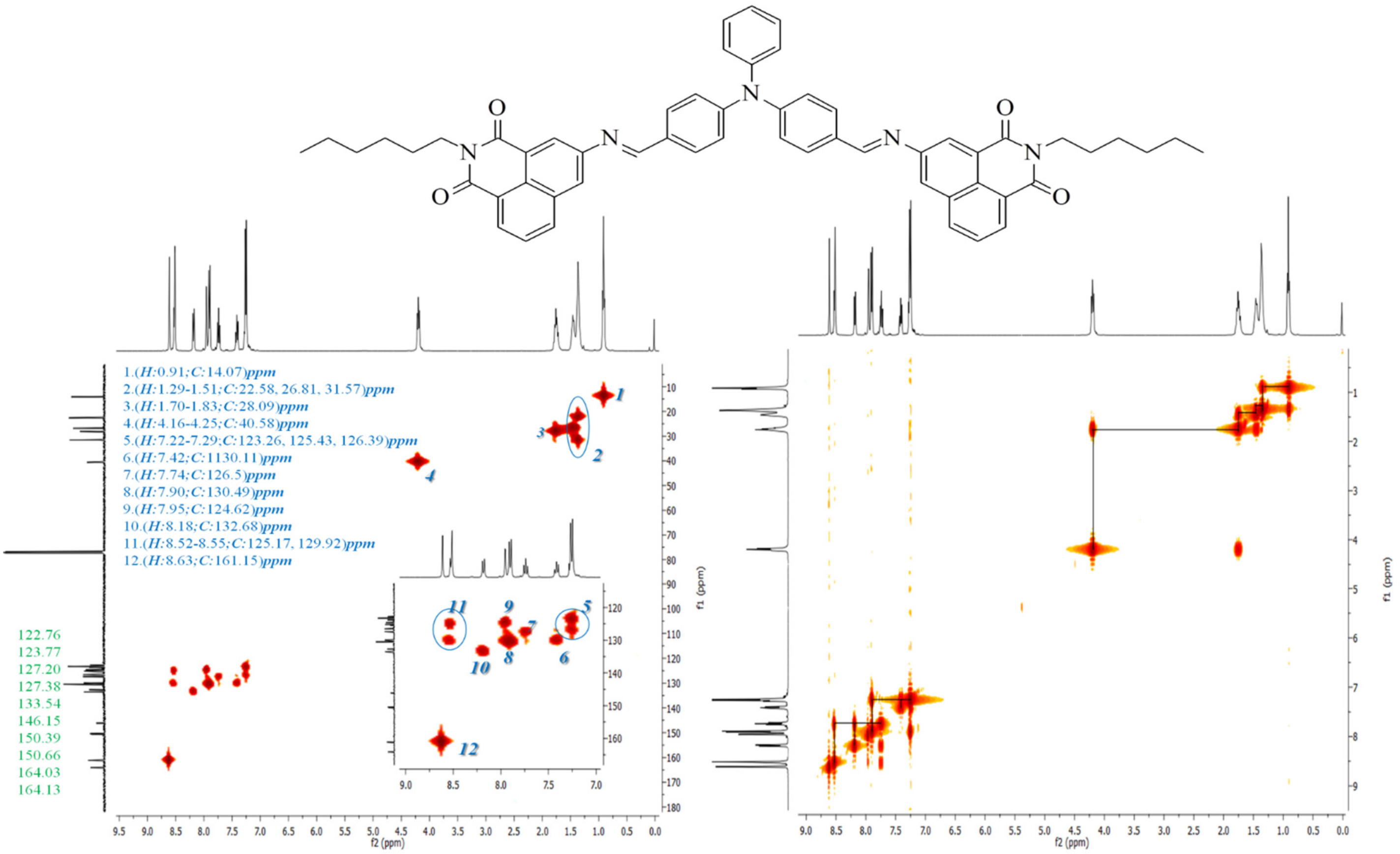

Figure S4. HMQC and COSY correlation spectra for 1a (400 MHz, $CDCl_3$).

2. Synthesis of the 1,8-naphthalimides Derivatives

2.1. Synthesis of the 4,4′-ethyne-1,2-diyldibenzaldehyde

The procedure for the synthesis of 4-ethynylbenzaldehyde was described in an earlier work [48].Then, the thus obtained terminal alkyne (0.24 g, 1 mmol), 4-bromobenzaldehyde (0.19 g, 1 mmol), $Pd(PPh_3)_2Cl_2$ (60 mg), PPh_3 (40 mg), CuI (15 mg) were placed in a 25 cm^3 flask. 15 cm^3 of dried triethylamine was added and the flask was tightly closed with a septum cap. The reaction was carried out for 15 h at 60 °C in the presence of nitrogen. After this time, the triethylamine was evaporated and the product was purified by column chromatography using chloroform as an eluent.

Light yellow solid; Yield = 60%, ^1H NMR (δ, 400 MHz, $CDCl_3$, ppm): 10.06 (s, 2H), 7.92 (d, J = 8.3 Hz, 4H), 7.73 (d, J = 8.2 Hz, 4H), ^{13}C NMR (δ, 101 MHz, $CDCl_3$, ppm): 191.29, 135.93, 132.33, 129.64, 128.69, 92.11.

2.2. Synthesis of the bis-(imino-1,8-naphthalimide) Derivatives

3-Amino-N-hexyl/benzyl/4-methylbenzyl/2-phenethyl-1,8-naphthalimide were obtained by the method reported previously [1]. Synthesized 3-amino-1,8-naphthalimides (0.1 mmol) were dissolved in ethanol and stirred with 1 equivalent of appropriate dialdehyde: 4,4′-diformyltriphenylamine, 4,4′-biphenyldicarbaldehyde, 2,5-diformylthiophene, 4,4′-ethyne-1,2-diyldibenzaldehyde. The three drops of TFA to the reaction were added. The reactions were carried out at room temperature for 24 h. After that time the product was filtered and washed with EtOH. The final product were receive after dried in vacuum oven at 50 °C over 5 h.

3. The Investigated Compounds under Day Light and 1a under UV-light

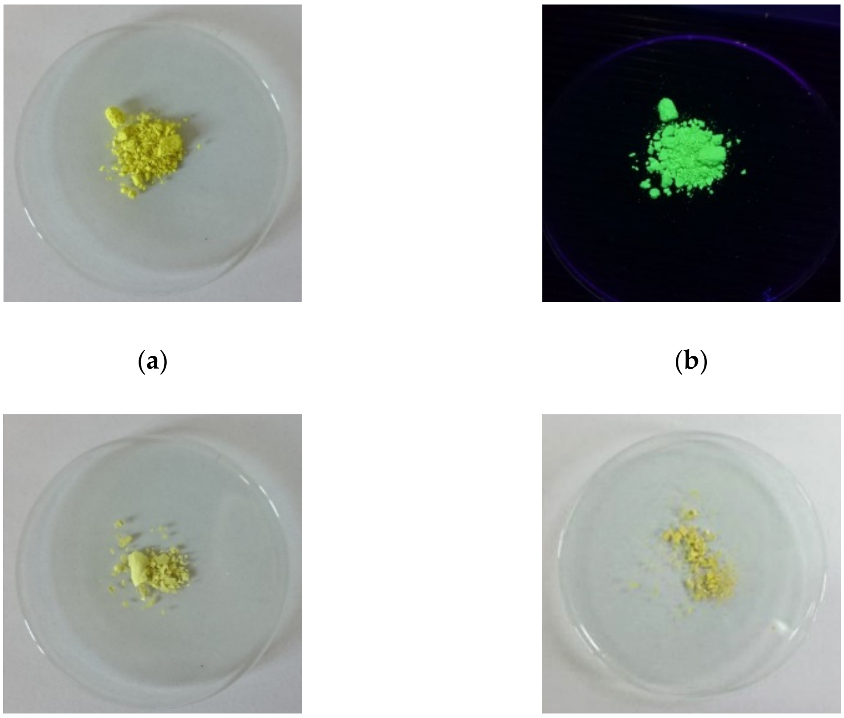

Figure S5. The investigated compounds under (**a**) 1a; (**b**) 1a under UV-light; (**c**) 1c; (**d**) 1d day light.

4. Film, Blends and Devices Preparation

The blends and films were prepared by spin-coating (1000 rpm, 60 s) on the glass substrates from a homogeneous chloroform solution (10 mg/mL). Moreover, the blends were prepared from the compound (2 or 15 in weight %)/PVK:PBD (50:50 in weight %) solutions. Finally, the films and blends were dried for 20 h in a vacuum oven at 55 °C.

The devices with configuration ITO/PEDOT:PSS/compound/Al and ITO/PEDOT:PSS/compound:PVK:PBD/Al with 2 wt.% of compound content in blend were prepared. Devices were prepared based on the OSSILA substrates with pixilated ITO anodes and properly cleaned before use. The ITO anodes were covered by PEDOT:PSS filmwith spin-coating at 5000 rpm for 60 s and annealed for 5 min at 120 °C. Active layer was spin-coated on top of the PEDOT:PSS layer from chloroform solution (10 mg/dm^3) at 1000 rpm for 60 s and annealed for 5 min at 100 °C. The Al electrode was vacuum-deposited at a pressure of $5·10^{-5}$ Torr. Electroluminescence spectra were measured with the voltage applied using a precise voltage supply (GwInstek PSP-405¸GW Instek, New Tapei, Taiwan) and the sample was fixed to an XYZ stage. Light from the OLED device was collected through a 30 mm lens, focused on the entrance slit (50 μm) of a monochromator (Shamrock SR-303i, Andor Technology, Belfast, UK) and detected using a CCD detector (AndoriDus 12305). Typical acquisition times were equal to 10 s. The pre-alignment of the setup was done using a 405 nm laser.

5. Characterization Methods

Nuclear magnetic resonance spectra were recorded on a Bruker AC400 spectrometer (Bruker, Berlin, Germany) in $CDCl_3$ as solvent and TMS as the internal standard. Using a Thermo Scientific Nicolet iS5 (Thermofisher, London, UK). The elementary analysis was measured using Vario EL III apparatus (Elementar, Langenselbold, Germany). Thermal instigations were performed using a Du Pont 1090B apparatus (Dupont, Wilmington, DE, USA) with a heating/cooling rate of 20 $°C·min^{-1}$ under nitrogen and using aluminum

sample pans in the range of 0–250 °C and a Mettler Toledo TGA STARe (Mettler Toledo, Warszawa, Poland) system with a heating rate of 10 °C·min^{-1} in a constant stream of nitrogen (20 mL·min^{-1}) and a temperature range from 50 to 800 °C. The glass transition temperature (T_g) was recorded in the second heating scan after first heating scan and cooling scan. Absorption spectra were performed using an Evolution 220 UV-Visible Spectrophotometer (Thermofisher, London, UK) with 1 cm quartz cell and Jasco V-550 Spectrophotometer (Jasco, Easton, MD, USA) for films and blends. The Varian Carry Eclipse Spectrometer (Santa Clara, US) was used to recorded photoluminescence spectra in solutions and the Hitachi F-2500 Spectrometer (Hitachi, Berkshire, UK) for blends and films photoluminescence measurements. Quantum yields (Φ_{PL}) measurements were performed by using the integrating sphere AvantesAvaSphere-80 (Edinburgh Instruments, Austion, TX, US) and absolute method. The lifetime (τ) of photoluminescence was measured with a time-correlated single photon counting (TCSPC). The time-resolvedmeasurements were performed using the picosecond pulsed diode laser, EPL-340 nm and laser EPL-45 nm, using a 60 W microsecond Xe flash lamp. Pulse period for all measurements was equal to 50 ns and PMT (Hamamatsu R928P, Hamamatsu Photonics, Iwata, Japan) as a detector. The fluorescence decay analysis was received an instrument response function (IRF) using ludox solution and results were presented as average values of decay after exponential fitting. Electrochemical measurements (CV and DPV) were performed with Eco ChemieAutolab PGSTAT128n (Metrohm Autolab B.V., Ulrecht, The Netherlands) potentiostat in a one-compartment cell in CH_2Cl_2 (Sigma-Aldrich, Saint Louis, MS, USA, for HPLC, 99.8%). A platinumwire (diameter 2.0 mm) served as a working electrode. The platinum coil and silver wire were used as auxiliary and reference electrode, respectively. As the supporting electrolyte salt the 0.1 mol/dm^3 Bu_4NPF_6 (Sigma-Aldrich, Saint Louis, MS, USA, 99%) was used. Each experiment was performed in an air-conditioned room at 20 ± 1 °C and under argon purging. The measurements were recorded with moderate scan rate equal to 0.1 V/s for CV and 0.01 V/s for DPV. All potentials were referenced to the stable Fc/Fc^+ couple (and the IP of ferrocene couple was calculated to be equal to −5.1 eV) [2].

6. DFT Calculations

Theoretical calculations were performed with the use of the density functional theory (DFT) and were carried out using the Gaussian09 program [3] on B3LYP/6-311g++ level [4]. Molecular geometry of the singlet ground state of the compounds was optimized in the gas phase and the frequency calculation for each of the compounds shows only positive values which verify that the optimized molecular structure corresponds to energy minimum. Solvent effect was taken into account using polarizable continuum model (PCM) [5] with dichloromethane and chloroform as solvents. Such calculations were carried out for analysis of the frontier molecular orbitals structure, energy levels and UV-Vis data. The optimized geometries of the compounds are depicted in Figure S1. Density of states diagram were obtained with use of GaussSum program [6]. The TD-DFT (time dependent density functional theory) method [7] was employed to calculate the electronic absorption spectrum of the compounds in chloroform. Experimental and calculated UV-vis spectra are presented in the Figure S11.

7. DSC Thermograms

(**a**)

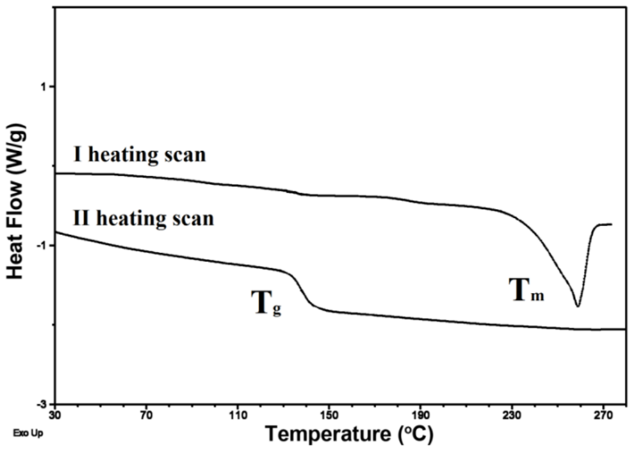

(**b**)

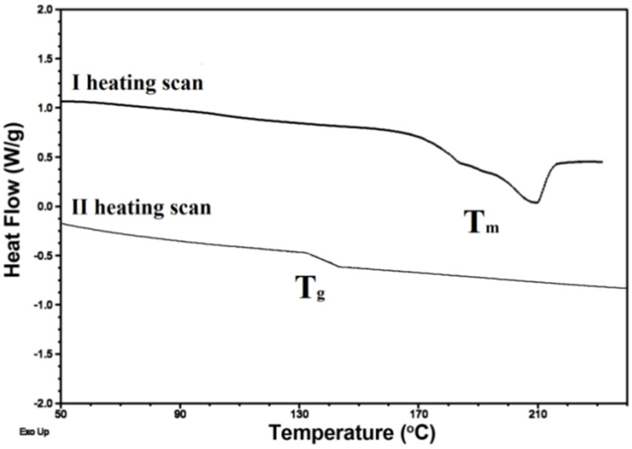

(**c**)

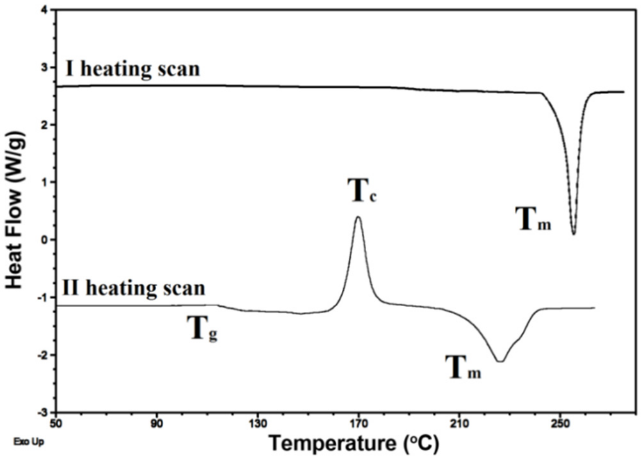

Figure S6. DSC thermograms of (**a**) 2a; (**b**) 3a and (**c**) 4b.

8. The (A) Reduction and (B) Oxidation Processes of 2a, 3a, 4b

(**a**)

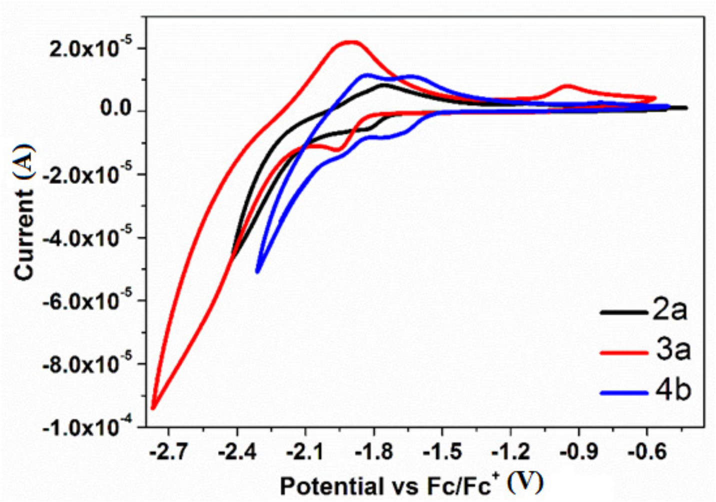

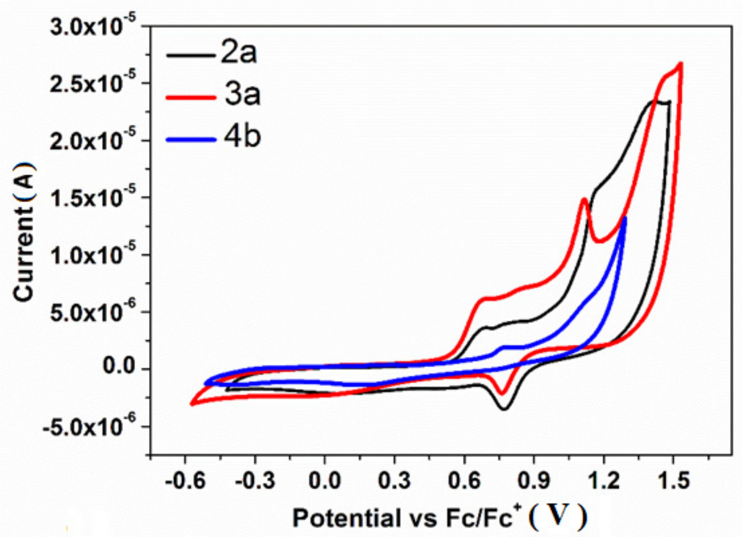

(**b**)

Figure S7. The (**a**) reduction and (**b**) oxidation processes of 2a, 3a, 4b (Pt, v= 0.1 V/s, 0.1 mol/dm^3 Bu_4NPF_6 in CH_2Cl_2 with 10^{-3} mol/dm^3 of compounds).

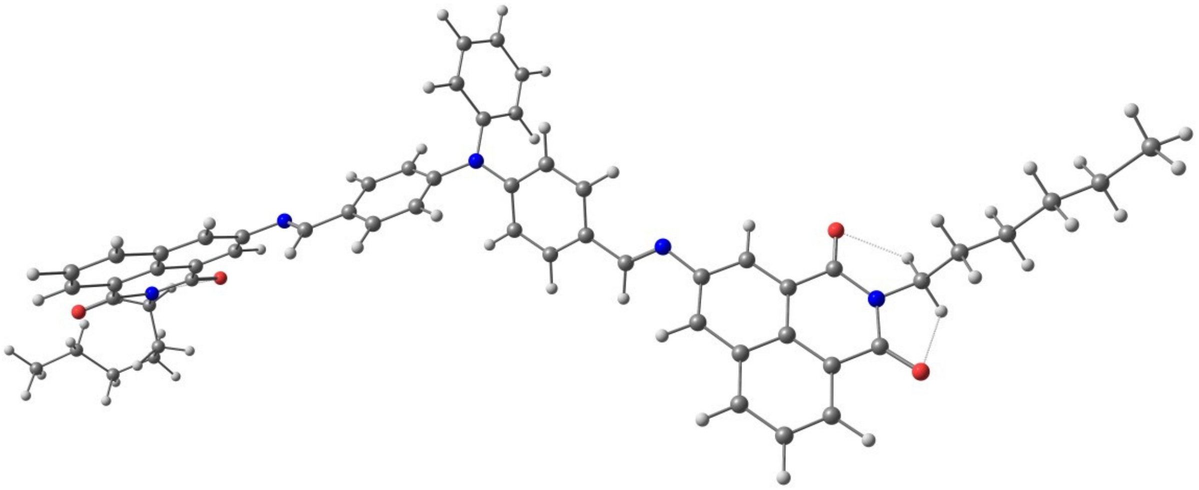

(**a**)

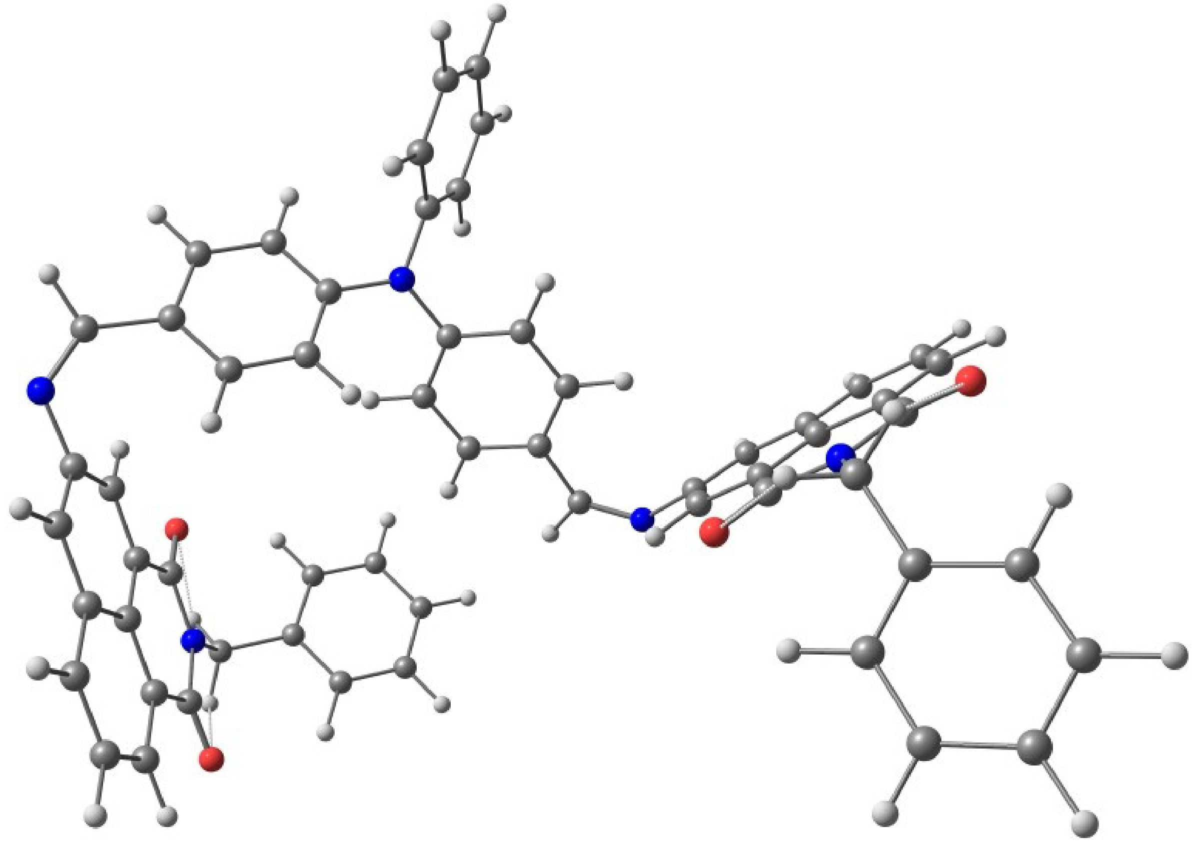

(**b**)

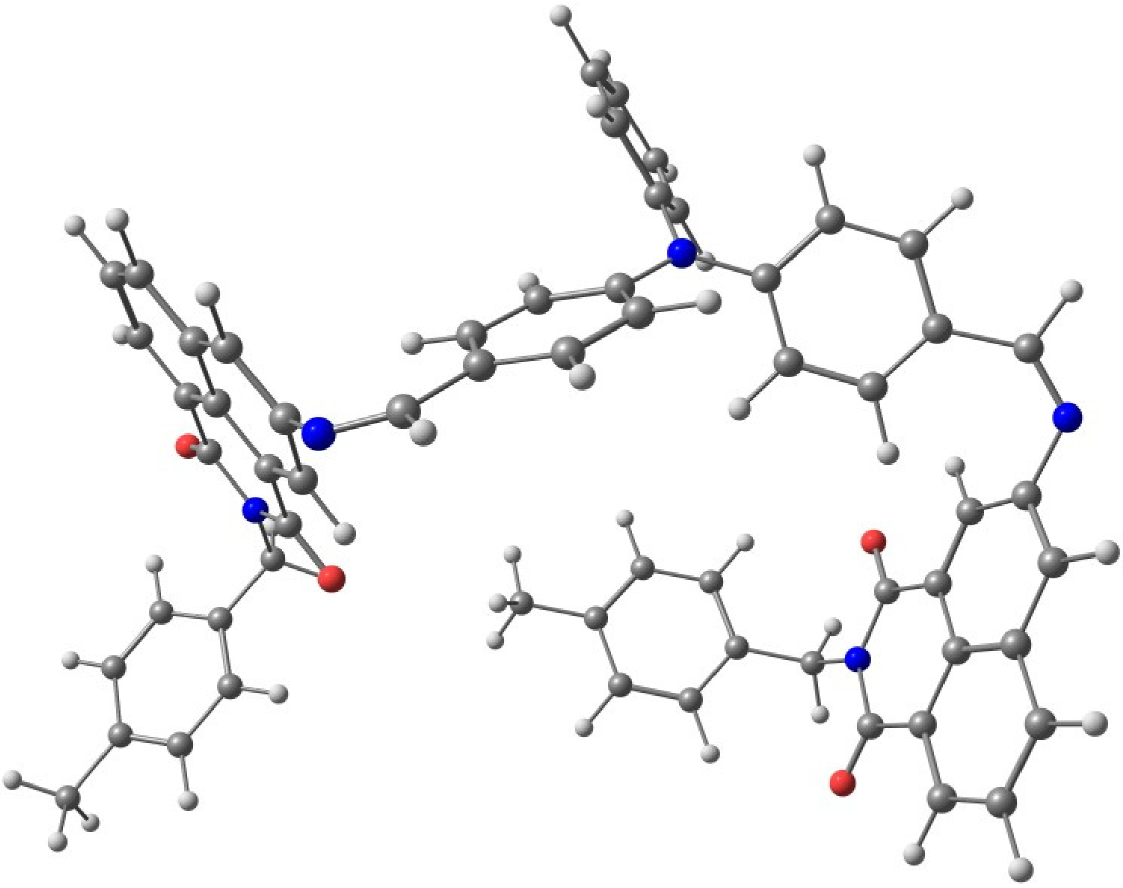

(**c**)

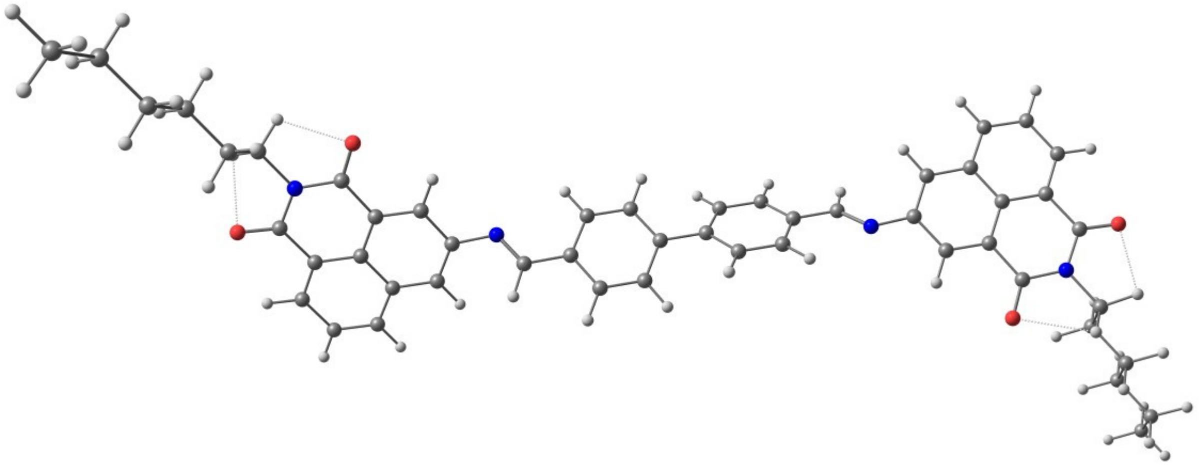

(**d**)

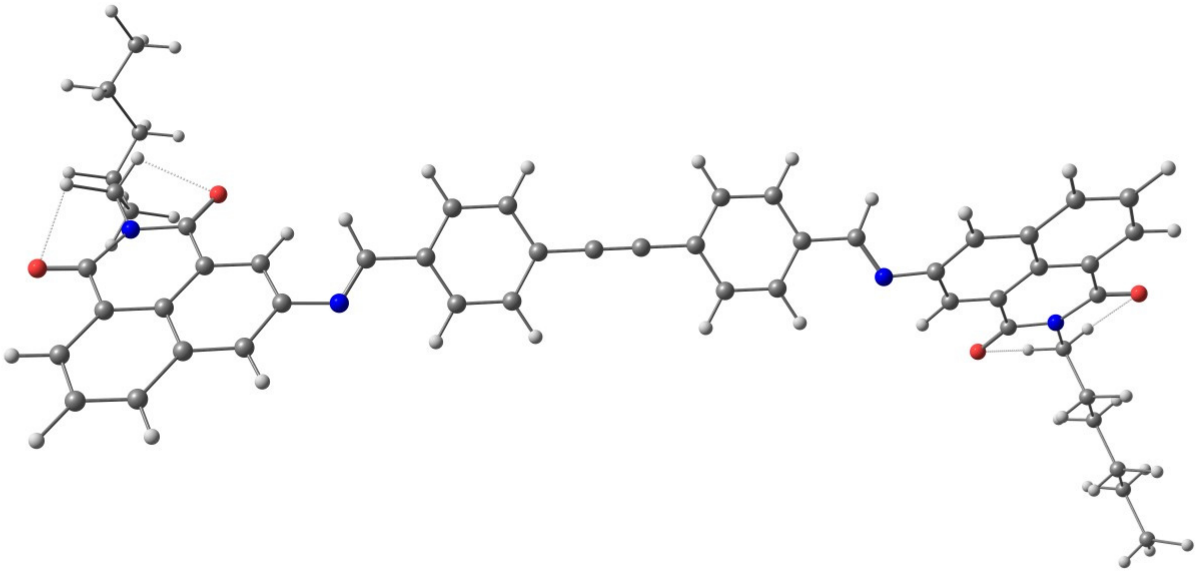

(**e**)

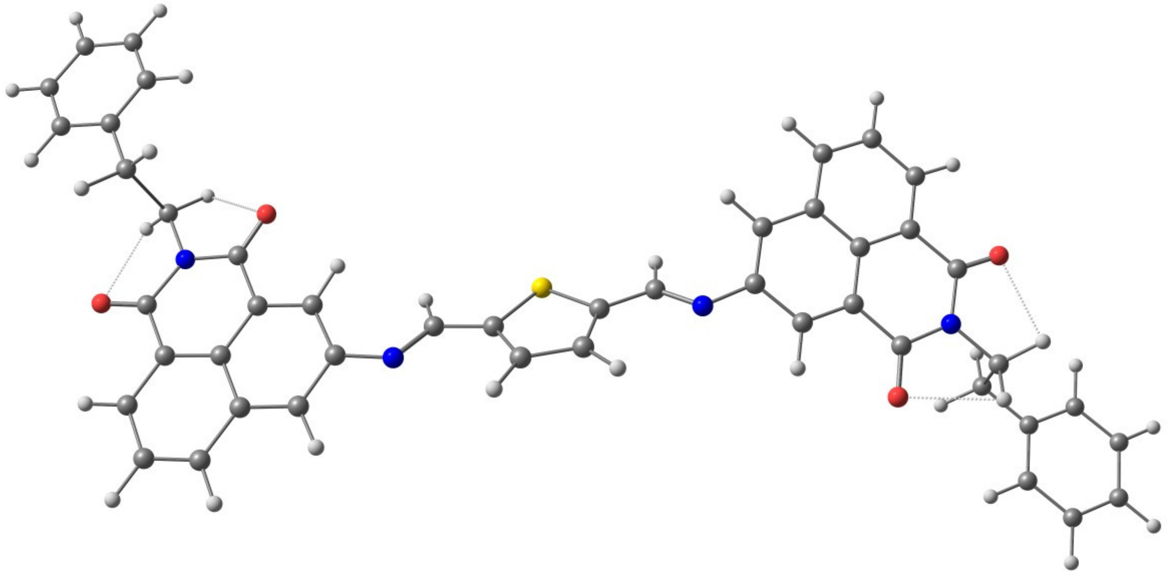

(**f**)

Figure 8. Optimized geometries of the compounds (**a**) 1a; (**b**) 2a; (**c**) 3a; (**d**) 1c; (**e**) 1d; (**f**) 4b.

HOMO LUMO

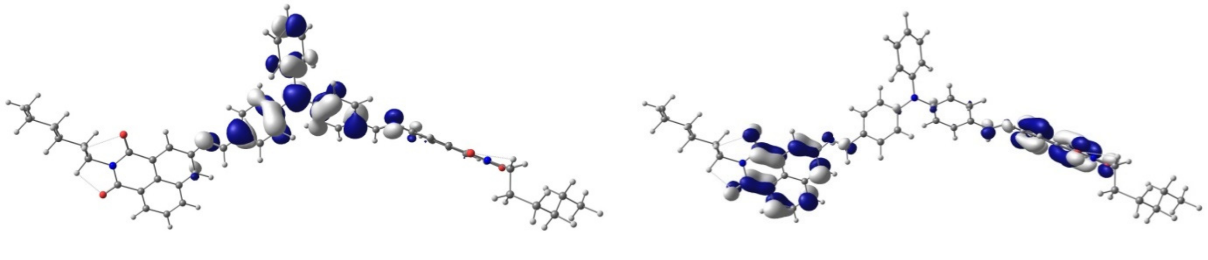

(**a**)

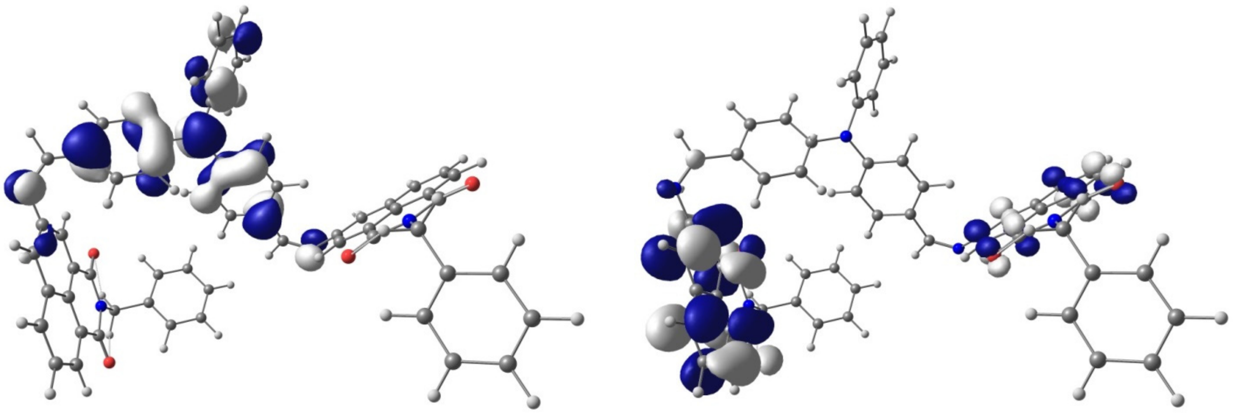

(**b**)

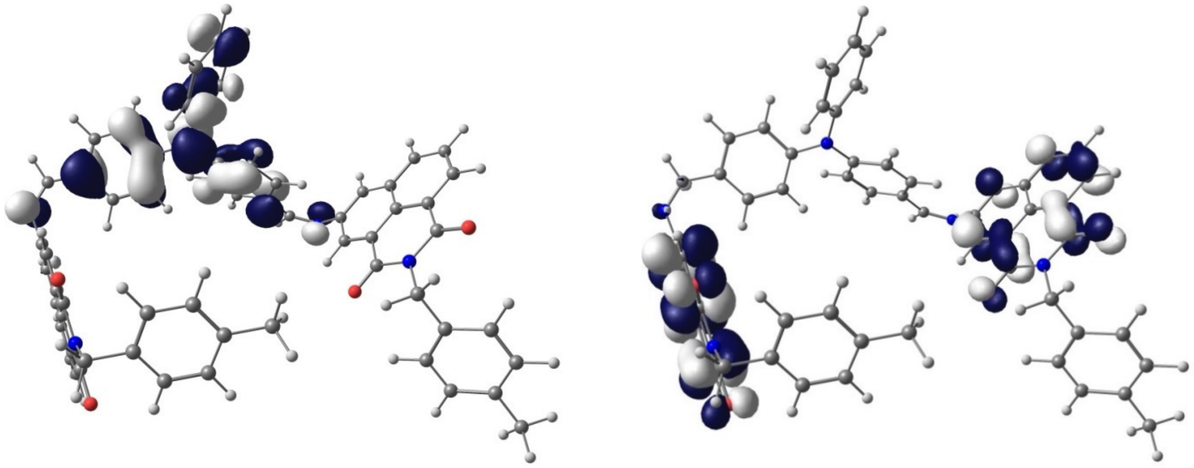

(**c**)

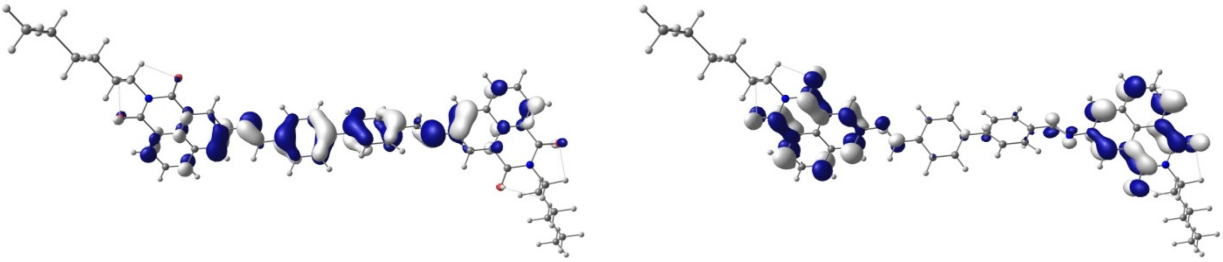

(**d**)

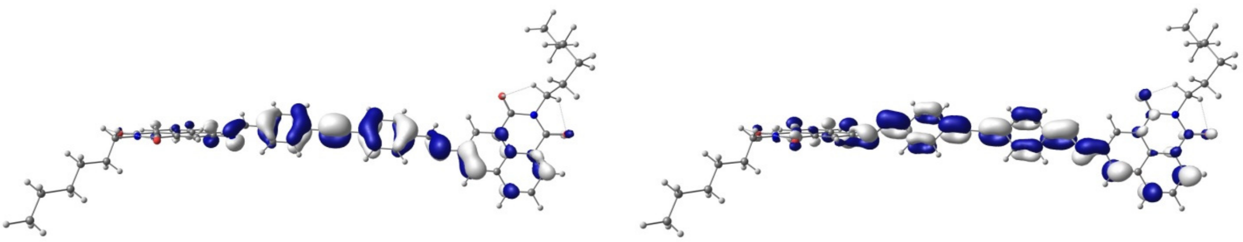

(**e**)

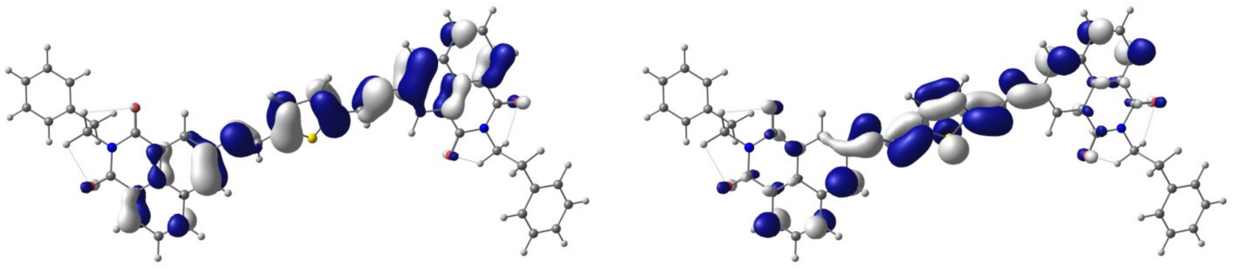

(**f**)

Figure 9. Contours of HOMO and LUMO of the compounds (**a**) 1a; (**b**) 2a; (**c**) 3a; (**d**) 1c; (**e**) 1d; (**f**) 4b.

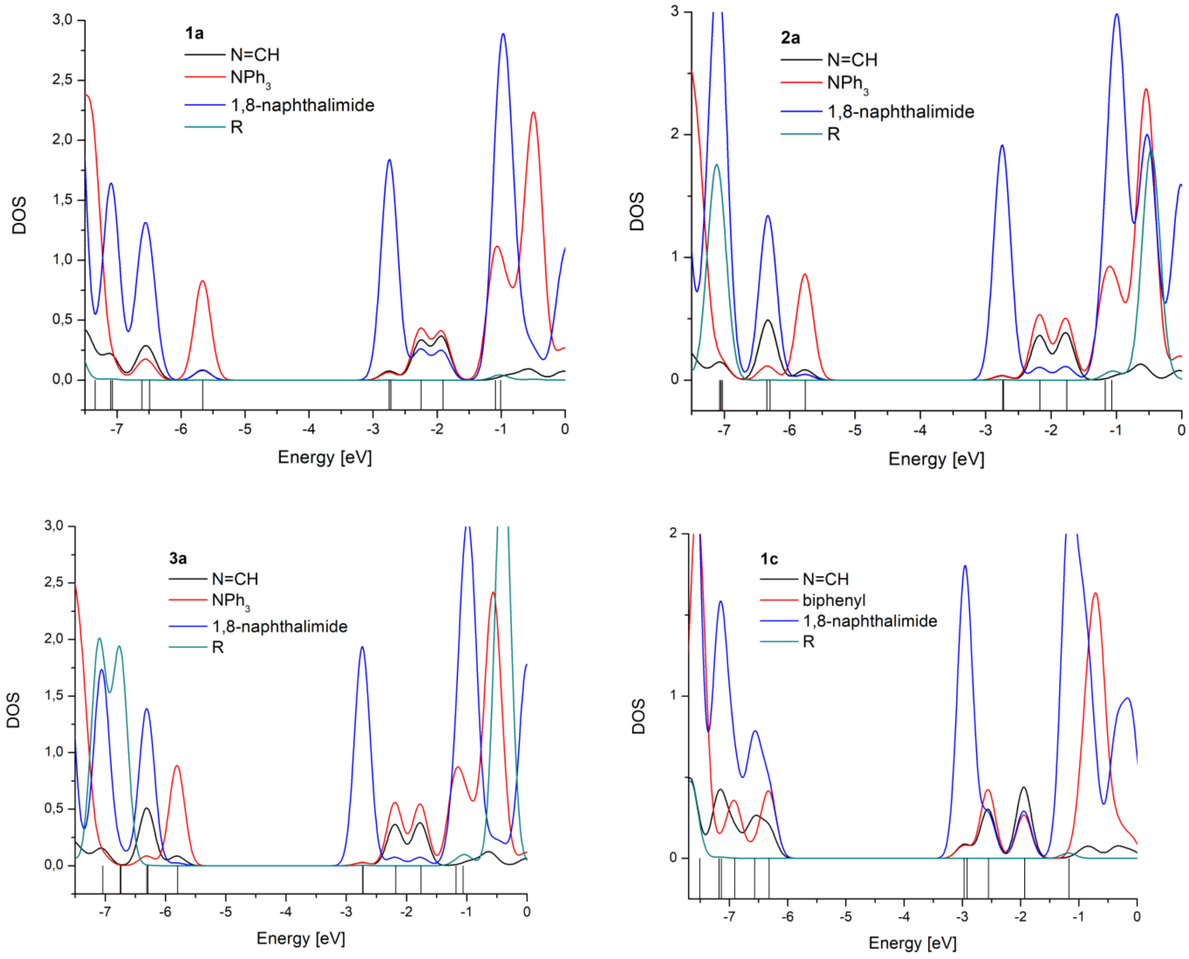

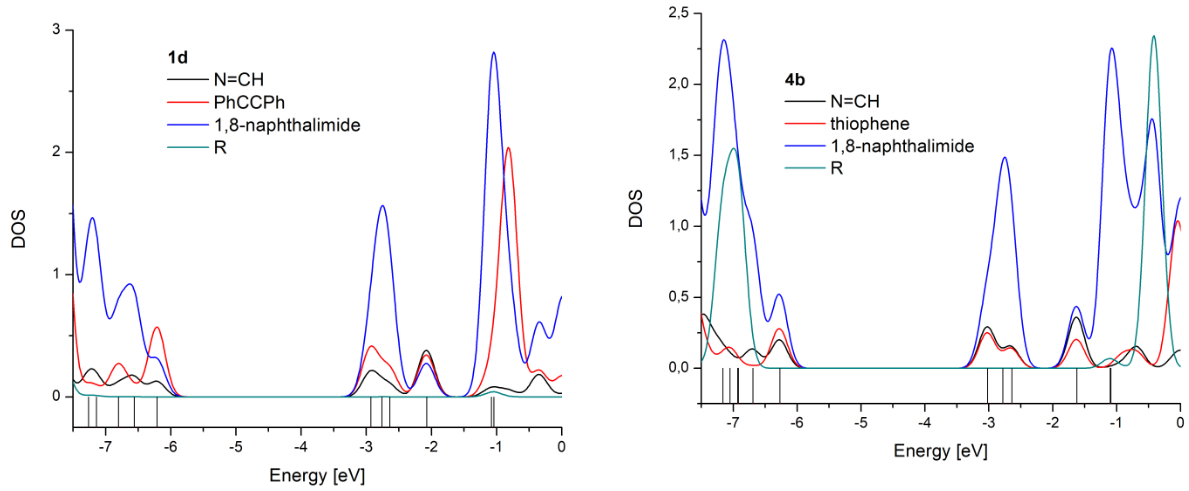

Figure S10. Density-of-states diagrams of ground state of the molecules.

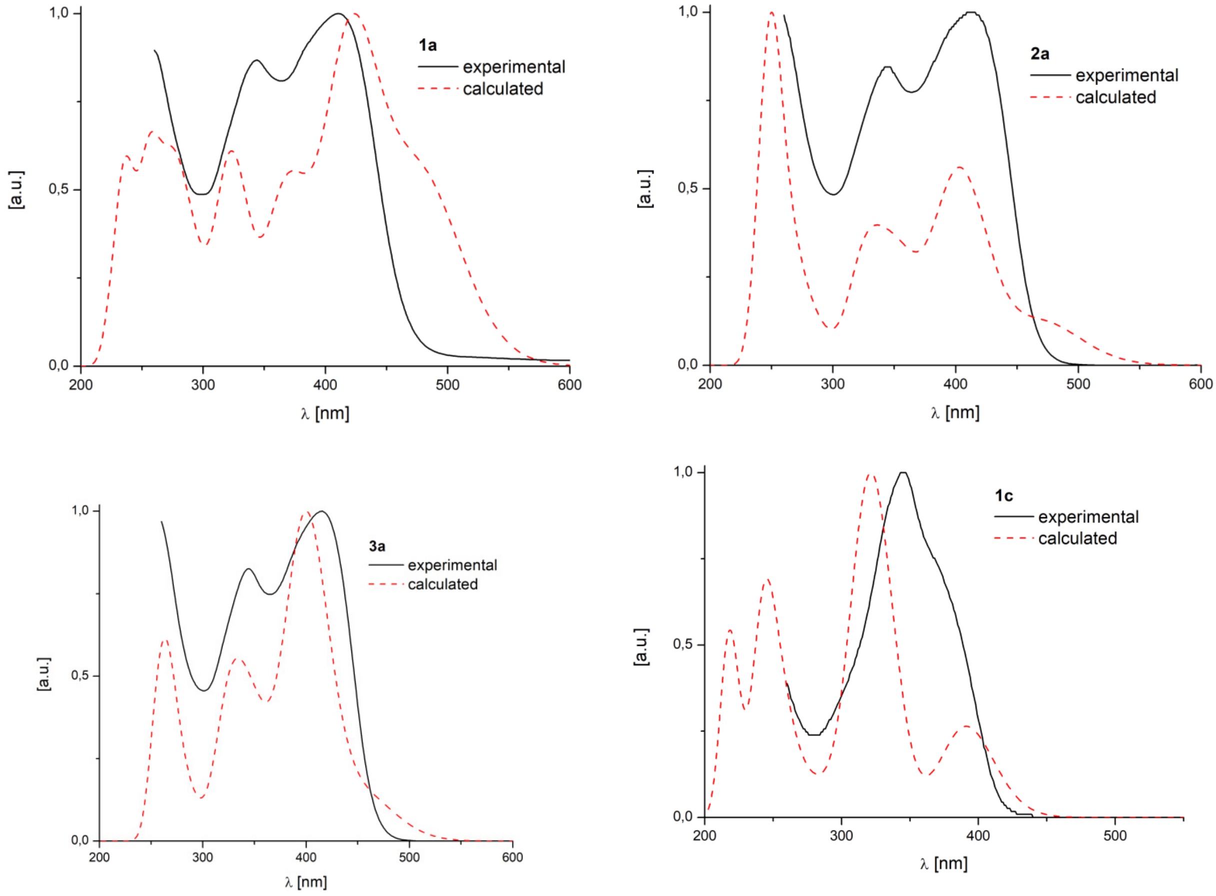

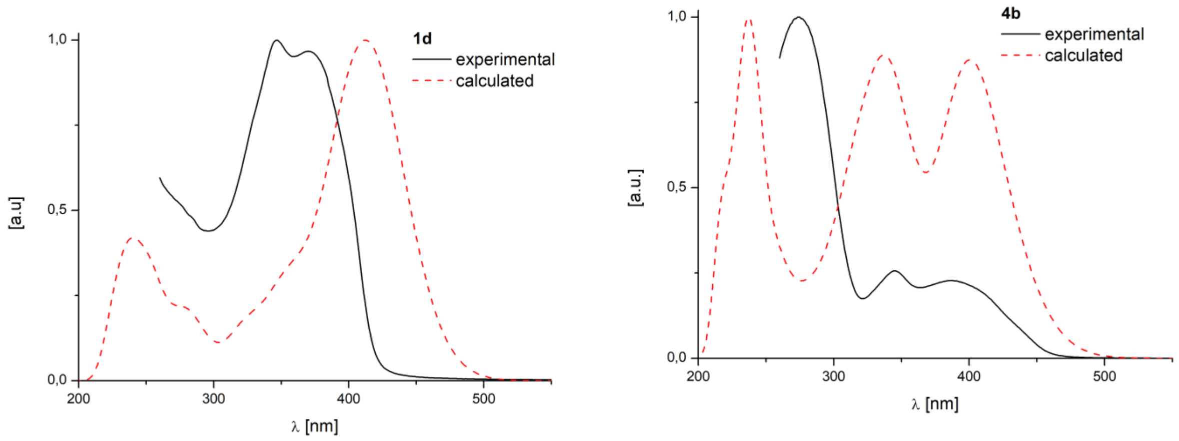

Figure S11. Experimental and calculated UV-Vis spectra of bis-(imino-1,8-naphthalimide) derivatives.

Table S2. Calculated dipole moments (in $CHCl_3$) and geometrical parameters of the molecules in ground and first singlet excited state.

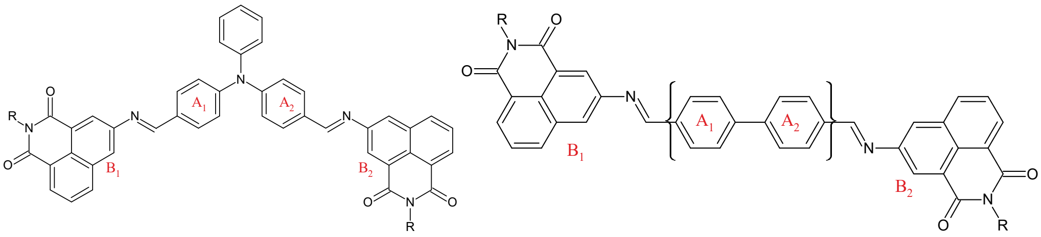

	**∠ A_1–B_1 [°]**	**∠ A_2–B_2 [°]**	**∠ B_1–R [°]**	**∠ B_2–R [°]**
		S_0		
1a	46.30	47.25	45.61	89.68
2a	65.09	73.31	63.07	66.42
3a	68.85	83.79	68.31	66.99
1c	44.10	43.60	89.49	89.68
1d	46.59	34.95	89.26	89.71
4b	33.14	44.78	2.81	3.21
		S_1		
1a	2.39	37.77	44.98	89.46
2a	58.67	61.89	59.51	66.73
3a	55.16	68.06	66.15	65.18
1c	24.81	27.22	89.26	89.71
1d	19.50	23.99	89.33	89.54
4b	22.24	24.33	2.81	3.21

Table S3. Composition of the selected MO in ground state of the compounds.

**1a**	**eV**	**N = CH**	**TPA**	**NDI**	**R**	**2a**	**eV**	**N = CH**	**TPA**	**NDI**	**R**
L + 5	−1.01	0	0	97	2	L + 5	−1.07	0	4	92	4
L + 4	−1.09	0	96	3	0	L + 4	−1.17	0	65	34	1
L + 3	−1.91	36	40	24	0	L + 3	−1.76	39	50	11	0
L + 2	−2.25	32	42	25	0	L + 2	−2.17	36	53	10	0
L + 1	−2.72	2	2	96	0	L + 1	−2.73	1	1	97	0
LUMO	−2.75	5	5	90	0	LUMO	−2.74	2	3	95	0
HOMO	−5.66	9	83	8	0	HOMO	−5.76	9	87	5	0
H − 1	−6.49	17	10	73	0	H − 1	−6.30	26	4	70	0
H − 2	−6.61	15	10	75	0	H − 2	−6.35	24	8	68	0
H − 3	−7.07	8	8	84	0	H − 3	−7.03	6	8	75	12
H − 4	−7.10	13	5	82	1	H − 4	−7.05	2	2	30	66
H − 5	−7.34	7	88	5	0	H − 5	−7.07	7	3	72	18
**3a**	**eV**	**N = CH**	**TPA**	**NDI**	**R**	**1c**	**eV**	**N = CH**	**Biphenyl**	**NDI**	**R**
L + 5	−1.06	0	5	91	5	L + 5	−1.17	0	0	98	2
L + 4	−1.18	0	71	28	1	L + 4	−1.17	0	0	98	2
L + 3	−1.76	38	55	7	0	L + 3	−1.93	44	27	29	0
L + 2	−2.18	37	56	8	0	L + 2	−2.55	30	42	27	0
L + 1	−2.72	1	1	98	0	L + 1	−2.92	2	1	97	0
LUMO	−2.73	2	2	96	0	LUMO	−2.97	7	7	86	0
HOMO	−5.80	9	89	2	0	HOMO	−6.32	18	41	41	0
H − 1	−6.29	26	3	71	0	H − 1	−6.57	23	6	71	0
H − 2	−6.31	25	5	69	0	H − 2	−6.91	16	35	49	0
H − 3	−6.74	0	0	6	94	H − 3	−7.14	14	2	84	0
H − 4	−6.75	0	0	5	95	H − 4	−7.18	26	5	68	0
H − 5	−7.04	7	6	75	13	H − 5	−7.51	2	97	1	0
**1d**	**eV**	**N = CH**	**PhCCPh**	**NDI**	**R**	**4b**	**eV**	**N = CH**	**Thiophene**	**NDI**	**R**
L + 5	−1.04	0	1	97	2	L + 5	−1.09	0	0	95	4
L + 4	−1.08	5	17	77	0	L + 4	−1.10	0	0	97	3
L + 3	−2.07	38	34	27	0	L + 3	−1.62	36	20	44	0
L + 2	−2.64	9	22	69	0	L + 2	−2.64	15	14	72	0
L + 1	−2.76	3	2	95	0	L + 1	−2.78	1	1	98	0
LUMO	−2.93	20	39	42	0	LUMO	−3.02	29	25	46	0
HOMO	−6.21	13	57	30	0	HOMO	−6.27	20	28	52	0
H − 1	−6.56	16	7	77	0	H − 1	−6.69	13	2	85	0
H − 2	−6.80	10	26	64	0	H − 2	−6.92	0	0	7	93
H − 3	−7.14	11	4	84	1	H − 3	−6.93	0	0	65	35
H − 4	−7.26	14	8	77	1	H − 4	−7.05	7	14	77	2
H − 5	−7.63	5	8	86	1	H − 5	−7.16	0	0	0	100

Table S4. The calculated electronic transitions corresponding to excitation resulting most intense luminescence in $CHCl_3$ solution.

	λ_{exp}	λ_{calc} (f)[#]	Transition	Character
1a	340	341.3 (0.0716)	H - 2 → L + 1 (52%); H - 1 → LUMO (18%)	$\pi_{NDI} \rightarrow \pi_{NDI}^*$
2a	340	347.0 (0.1483)	H - 2 → L + 2 (27%); HOMO → L + 3 (55%)	$\pi_{NDI/\text{-}N=CH\text{-}} \rightarrow \pi_{TPA}^*$; $\pi_{TPA} \rightarrow \pi^*$
3a	340	346.0 (0.2454)	HOMO → L + 3 (89%)	$\pi_{TPA} \rightarrow \pi_{NDI/\text{-}N=CH\text{-}}^*$
1c	340	330.1 (0.1596)	H - 2 → LUMO (62%); H - 2 → L + 1 (10%)	$\pi \rightarrow \pi_{NDI}^*$

1d	340	355.7 (0.4467)	H - 2 → LUMO (86%)	$\pi \rightarrow \pi^*$
4b	340	341.0 (0.0979)	H - 2 → LUMO (43%); H - 4 → LUMO (22%); H - 3 → LUMO (16%)	$\pi_R \rightarrow \pi^*$; $\pi_{NDI} \rightarrow \pi^*$; $\pi_{NDI/R} \rightarrow \pi^*$

into account were taken the calculated transitions at the wavelength closest to the experimental data with the highest transition coefficient in this energy range.

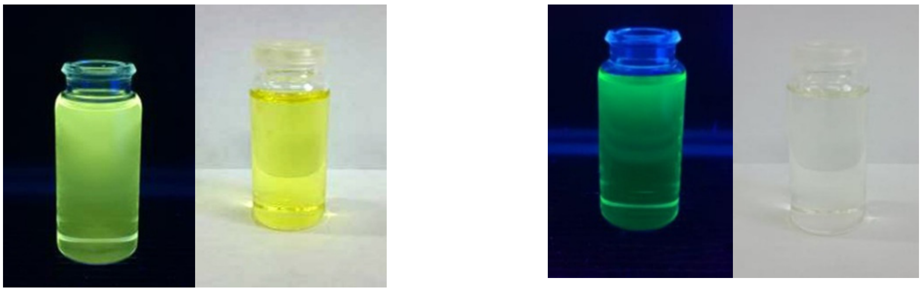

(**a**) (**b**)

Figure S12. (**a**) 1a and (**b**) 1d under day light and under UV-light (λ = 366 nm) in NMP.

Table S5. PL life-time (τ) measurements data.

Code	Medium	τ_1 [ns] (Share %)	τ_2 [ns] (Share %)	∑τ [ns]	χ^2
**1a**	Chloroform	0.12 (24.52)	16.78 (75.48)	16.9	1.045
	Film	3.63 (84.1)	8.83 (15.9)	12.5	0.930
**2a**	Chloroform	2.61 (23.2)	16.46 (76.8)	19.1	1.017
	Film	4.78 (92.36)	11.38 (7.64)	16.2	1.061
**3a**	Chloroform	2.31 (14.52)	13.81 (85.48)	21.0	1.063
	Film	4.22 (89.92)	11.08 (10.08)	15.3	1.098
**4b**	Chloroform	1.98 (6.3)	19.97 (93.7)	22.0	0.935
	Film	0.46 (69.05)	1.7 (30.95)	2.16	1.019
**1c**	Chloroform	0.36 (36.89)	16.34 (63.11)	16.7	1.123
	Film	2.34 (59.69)	8.56 (40.31)	10.9	1.056
**1d**	Chloroform	1.37 (46.48)	9.83 (53.52)	11.2	1.012
	Film	1.84 (62.45)	7.12 (37.55)	8.96	1.047

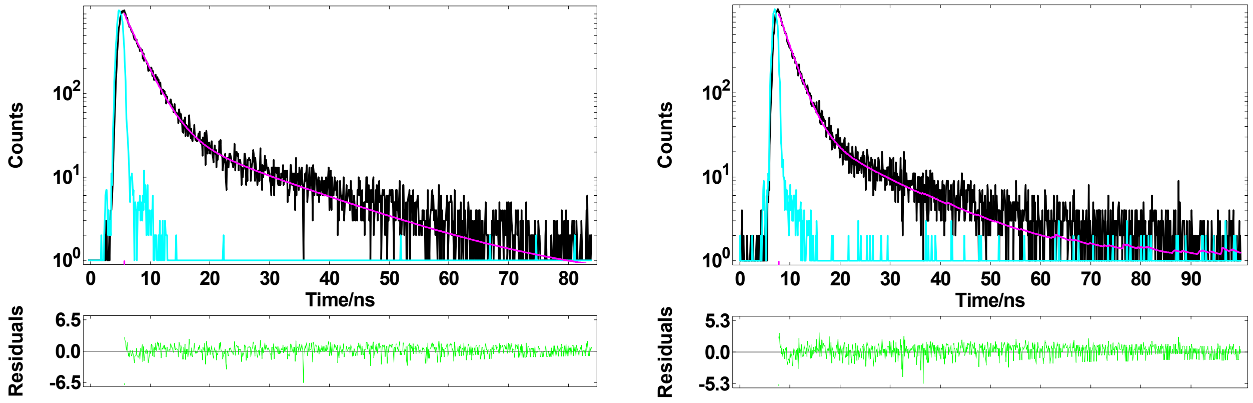

(**a**) (**b**)

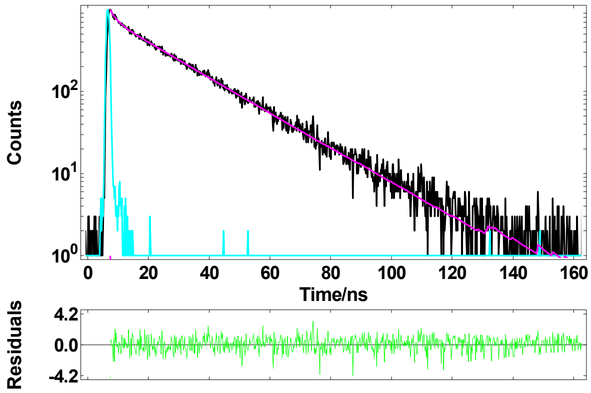

(**c**)

Figure S13. PL life-time (τ) curves of (**a**) 2a; (**b**) 3a and (**c**) 4b in chloroform solution.

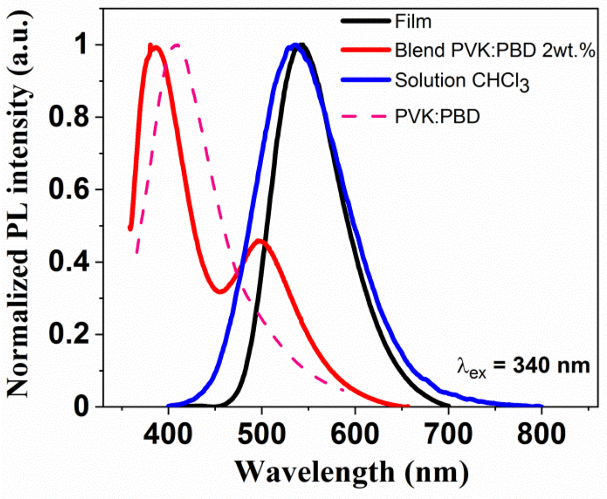

(**a**)

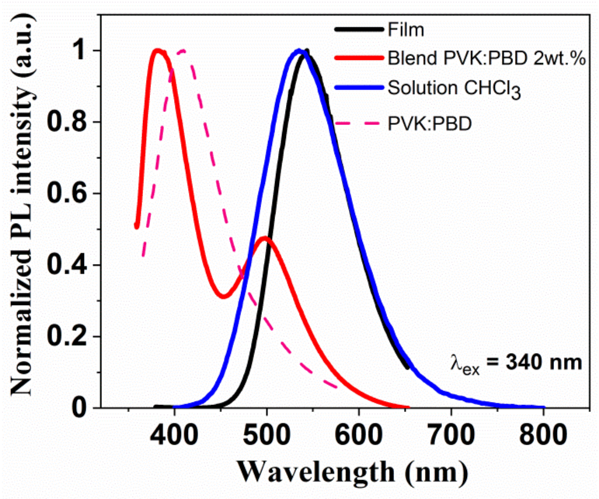

(**b**)

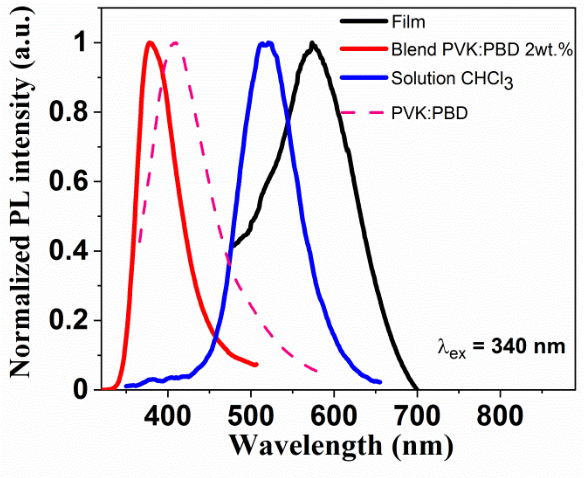

(**c**)

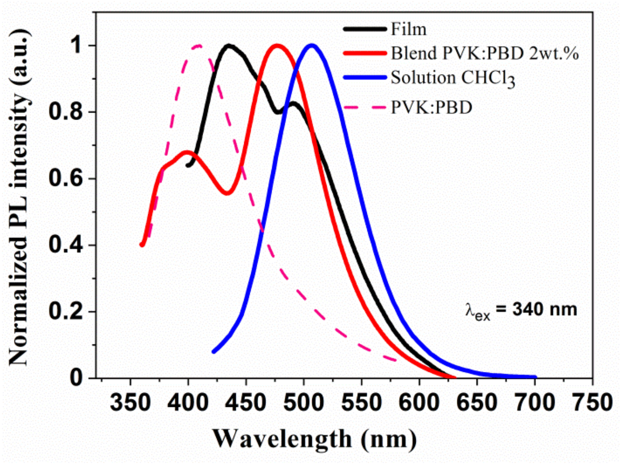

(**d**)

Figure S14. Emission spectra of (**a**) 2a; (**b**) 3a; (**c**) 4b and (**d**) 1c with PVK:PBD.

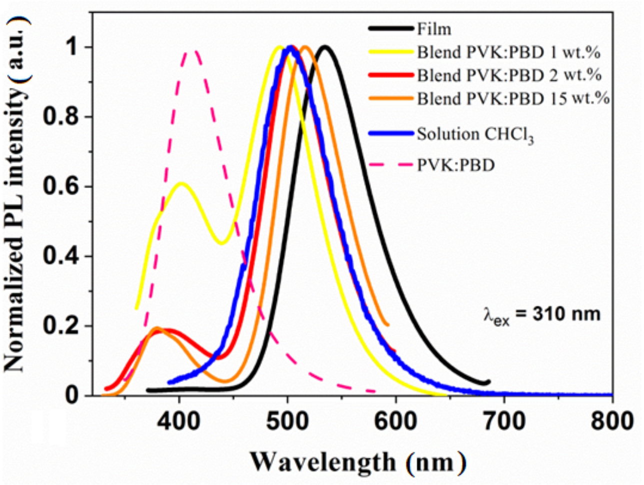

Figure S15. Emission spectra of 1a and PVK:PBD.

Table S6. EA, IP, HOMO, LUMO, E_g and E_g^{opt}.

Compound	Method	EA [eV]	IP [eV]	HOMO [eV]	LUMO [eV]	E_g [eV]	E_g^{opt} [eV]
1a	CV [a]	−3.87	−5.63	-	-	1.76	-
	DPV [a]	−3.88	−5.46	-	-	1.58	-
	DFT	-	-	−5.66	−2.75	2.91	-
	Solution [b]	-	-	-	-	-	2.63
2a	CV [a]	−3.37	−5.65	-	-	2.28	-
	DPV [a]	−3.33	−5.63	-	-	2.30	-
	DFT	-	-	−5.78	−2.74	3.01	-
	Solution [b]	-	-	-	-	-	2.62
3a	CV [a]	−3.27	−5.66	-	-	2.39	-
	DPV [a]	−3.22	−-5.58	-	-	2.36	-
	DFT	-	-	−5.80	−2.73	3.08	-
	Solution [b]	-	-	-	-	-	2.62
4b	CV [a]	−3.35	−5.56	-	-	2.21	-
	DPV [a]	−3.43	−5.66	-	-	2.23	-
	DFT	-	-	−6.27	−3.02	3.25	-
	Solution [b]	-	-	-	-	-	2.71
1c	CV [a]	−3.72	−5.51	-	-	1.79	-
	DPV [a]	−3.82	−5.48	-	-	1.66	-
	DFT	-	-	−6.32	−2.92	3.35	-
	Solution [b]	-	-	-	-	-	2.97
1d	CV [a]	−3.83	−5.8	-	-	1.97	-
	DPV [a]	−3.91	−5.8	-	-	1.89	-
	DFT	-	-	−6.21	−2.93	3.28	-
	Solution [b]	-	-	-	-	-	2.94

[a] Measurements in CH_2Cl_2. [b] Measurements ,in $CHCl_3$.

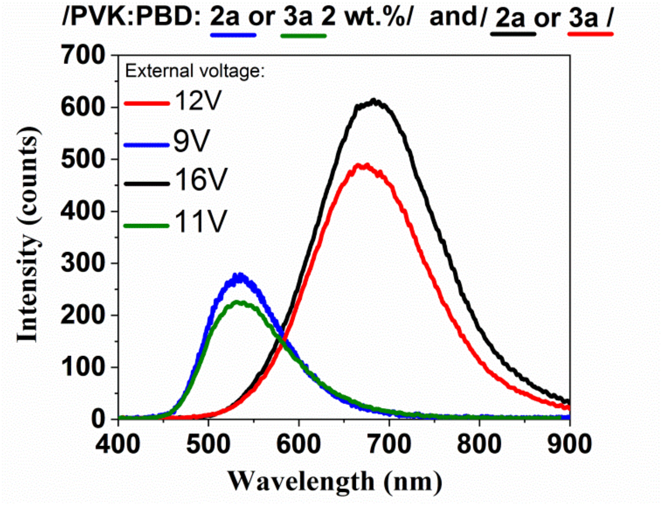

Figure S16. Electroluminescence (EL) spectra of the working devices under an applied voltage. Above the graphs the device structures are shown. Presented are maxima of EL intensity.

Reference

1. Kotowicz, S.; Korzec, M.; et.al. Novel 1,8-naphthalimides substituted at 3-C position: Synthesis and evaluation of thermal, electrochemical and luminescent properties. *Dye and Pigment.* **2018**, *158*, 65–78.
2. Bujak, P.; Kulszewicz-Bajer, I.; Zagorska, M.; et.al. Polymers for electronics and sprintronics. *Chem. Society Review* **2013**, *42*, 8895–8999.
3. Frisch, M.J.; Trucks, G.W.; Schlegel, H.B.; Scuseria, G.E.; Robb, M.A.; Cheeseman, J.R.; Scalmani, G.; Barone, V.; Petersson, G.A.; Nakatsuji, H.; et al., Gaussian, Inc., Wallingford CT, 2016.
4. Becke, A.D.; Density-functional thermochemistry. III. The role of exact exchange. *Journal of Chemical Physics* **1993**, *98*, 5648–5652.
5. Lee, C.; Yang, W.; Parr, R.G. Development of the colle-salvetti correlation-energy formula into a functional of the electron density. *Phys. review B* **1988**, *37*, 785–789.
6. O'Boyle, N.M.; Tenderholt, A.L.; Langner, K.M.; cclib: A library for package-independent computational chemistry algorithms. *J. of Computational Chemistry* **2008**, *29*, 839–845.
7. Casida, M.E. in: J.M. Seminario (Ed.), Recent Developments and Applications of Modern Density Functional Theory, *Theor. and Computational Chemistry*, Elsevier: Amsterdam, The Netherland, 1996, p. 391.
